# Pyrazolyl–Pyridine Ruthenium Complexes: A New Metallic Line of Defense Against *Acinetobacter baumannii*

**DOI:** 10.1002/cbic.70371

**Published:** 2026-05-10

**Authors:** M. Cassiem Joseph, Sheldon Sookai, Monika Nowakowska, Rotondwa Mphephu, Angela M. Kavanagh, Johannes Zuegg, Mark A. T. Blaskovich, Andrew J. Swarts

**Affiliations:** ^1^ School of Chemistry University of the Witwatersrand Johannesburg South Africa; ^2^ Department of Chemistry and Polymer Science University of Stellenbosch Stellenbosch South Africa; ^3^ Centre for Superbug Solutions Institute for Molecular Bioscience The University of Queensland St. Lucia Australia

**Keywords:** AMR, ESKAPE, inorganic medicinal chemistry, metallodrugs, metals in medicine

## Abstract

The emergence of antimicrobial resistance (AMR), especially among ESKAPE infections, highlights the pressing necessity for novel chemical frameworks with atypical modes of action. Transition‐metal complexes have emerged as attractive possibilities owing to their varied geometries, redox activity, and capacity to interact with biological sites inaccessible to traditional chemical antibiotics. This study presents the antibacterial and antifungal assessment of a series of Ru(II) pyrazolyl–pyridine half‐sandwich complexes (**C1**–**C6**), thereby broadening the chemical scope of metalloantibiotics previously investigated inside pyridyl–1,2,3‐triazolyl frameworks. The compounds were synthesized with good yields and structurally validated by single‐crystal X‐ray diffraction, demonstrating piano‐stool topologies with unique ligand‐dependent anion orientations. Extensive biological screening via the Community for Open Antimicrobial Drug Discovery (CO‐ADD) platform revealed selective efficacy of complexes **C1**–**C3** against Acinetobacter baumannii, a significant multidrug‐resistant pathogen. Cytotoxicity and hemolysis assessments revealed advantageous therapeutic ranges for the most potent combinations. Biophysical investigations, encompassing DNA‐binding fluorescence displacement, linear dichroism, and in silico molecular docking and dynamics, elucidated that the lead drug interacts with bacterial DNA via partial intercalation and groove contacts. Collectively, our findings establish Ru(II) pyrazolyl–pyridine complexes as prospective candidates for the advancement of next‐generation metalloantibiotics and underscore the significance of coordination‐chemistry‐driven approaches in addressing AMR.

## Graphical abstract

Ruthenium(II) (Ru(II)) pyrazolyl–pyridine half‐sandwich complexes (**C1–C6**) were synthesized and structurally characterized as prospective metalloantibiotics. Screening through the Community for Open Antimicrobial Drug Discovery (CO‐ADD) revealed selective activity against *Acinetobacter baumannii*, with favorable cytotoxicity profiles. Biophysical studies indicate partial DNA intercalation as a probable mode of action.

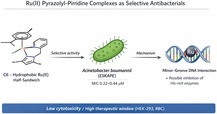

## 1 Introduction

The worldwide increase in antimicrobial resistance (AMR) is a critical threat to public health, compromising the effectiveness of current antibiotics and imposing significant strains on healthcare systems [1, 2, 3, 4, 5, 6]. Notwithstanding persistent endeavors, the clinical pipeline is inadequate, with few truly innovative therapeutic scaffolds arising in recent decades. The innovation gap is particularly alarming due to the rising incidence of multidrug‐resistant pathogens, specifically the ESKAPE group (Enterococcus faecium, Staphylococcus aureus, Klebsiella pneumoniae, Acinetobacter baumannii, Pseudomonas aeruginosa, and Enterobacter spp.), which constitute a significant percentage of life‐threatening infections globally [7, 8].

In response to these limitations, focus has shifted to unconventional chemical domains and treatment approaches. Transition metal complexes, historically utilized in cancer, catalysis, and imaging, are now gaining recognition as promising antimicrobial agents. Their distinctive three‐dimensional structures, variable oxidation states, and redox properties offer mechanisms of action unattainable by traditional chemical antibiotics. Systematic initiatives like the Community for Open Antimicrobial Drug Discovery (CO‐ADD) have evidenced that metal‐containing compounds exhibit significantly elevated hit rates against bacteria and fungi in comparison to solely organic molecules, frequently accompanied by low cytotoxicity and hemolysis, thereby highlighting their translational potential [9, 10, 11].

**SCHEME 1:**
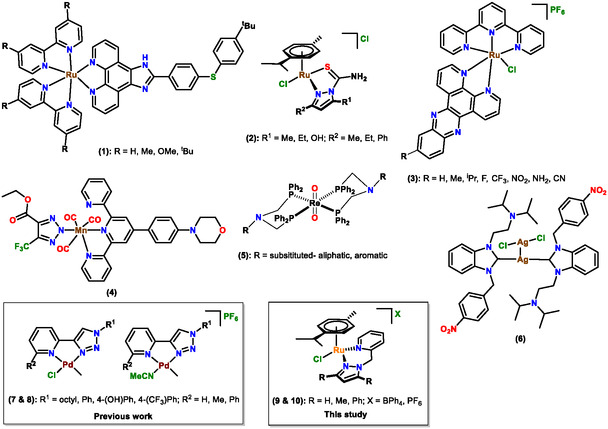
Transition metal complexes that displayed high levels of antimicrobial activity.

Ruthenium complexes have proven to be exceptionally adaptable scaffolds among the examined transition metals. Ru(II/III) polypyridyl complexes demonstrate robust DNA contacts, reactive oxygen species production, and membrane‐disruptive properties, facilitating multi‐target bactericidal effects [7, 12]. Derivatives containing aryl thioethers (1) or N, S donor ligands (2) exhibit significant efficacy against Gram‐positive bacteria, including methicillin‐resistant S. aureus, while demonstrating low toxicity in mammalian animals (Scheme 1) [12, 13].

Ruthenium terpyridine–dppz complexes (3) enhance this capability in photodynamic treatment (PDT), facilitating fast ROS‐mediated eradication of S. aureus both in vitro and in vivo [14].

PDT methods utilizing Ru and Ir photosensitizers constitute a resistance‐resilient strategy, as bacteria struggle to adapt to oxidative stress [15].

In addition to ruthenium, various metal‐based frameworks such as Mn(I) tricarbonyls (4), Re(V)‐oxo (5) and Ag(I)‐NHC (6) derivatives depicted in Scheme 1, have exhibited antibacterial or antifungal properties via multiple mechanisms, including membrane depolarization, DNA intercalation, metabolic inhibition, and reactive oxygen species generation [13, 16, 17, 18].

We recently reported the antimicrobial evaluation of Pd(II) complexes containing pyridyl–1,2,3‐triazolyl (pyta) ligands (7 and 8, Scheme 1), revealing significant antifungal efficacy in some Pd(II) analogs [19]. This underscored the essential function of ligand design in influencing biological efficacy and established a basis for investigating associated N‐donor frameworks.

In this context, the current study examines Ru(II) pyrazolyl–pyridine complexes (9 and 10(i.e., **C1‐C6**) as prospective antibacterial agents).

## 2 Experimental

### 2.1 Materials

All solvents (HPLC grade) and chemical synthons were used as received from Merck Sigma–Aldrich without further purification. Deuterated dimethyl sulfoxide was stored over activated 4 Å molecular sieves. Calf thymus DNA (ctDNA) was purchased from Sigma and used as received without further purification. Ultrapure water (Type I) was produced using a Merk‐Millipore Direct‐Q 3 UV Water Purification System. The preparation and characterization of the ligands were performed by a method previously reported [20]. The synthetic method is given in the ESI.

### 2.2 X‐Ray Crystal Structure Determination

Single crystal X‐ray diffraction data (SC‐XRD) for **C1–** **C3** were acquired using a Bruker Apex‐II CCD diffractometer with MoKα radiation (*λ* = 0.71073 Å) at a temperature of 173(2) K. Intensities were integrated using SAINT+ (v6.02) [21], and absorption corrections based on similar reflections were performed using SADABS [22]. Within the X‐Seed graphical user interface, all structures were solved using Direct Methods and then refined using full‐matrix least‐squares on F2 using SHELXS‐13 and SHELXL‐16 [23, 24, 25, 26]. Hydrogen atoms were positioned using riding models and computed positions, whereas all non‐hydrogen atoms were refined anisotropically. POV‐Ray was used to create high‐resolution molecular diagrams [27]. CSD 2 486 764 for **C1**, CSD 2 486 766 for **C2**, and CSD 2 486 765 for **C3** contain the supplementary crystallographic data for this paper.

### 2.3 Biological Evaluation

Full antimicrobial assessment was conducted by methods previously reported [28]. The full method is reported in the ESI [29].

### 2.4 Biophysical Chemistry

#### 2.4.1 pH Speciation

Spectroscopic $pK_{a}$ determinations and solution species characterization were performed using previously reported methods [30].

#### 2.4.2 DNA Binding Studies

Stock solution of calf thymus DNA (ctDNA) was prepared by dissolving the sodium salt (Sigma–Aldrich) in potassium phosphate buffer (pH 7.5, 50 mM) and slowly agitating it overnight at 140 rpm. The concentration of the solution was determined spectrophotometrically (*ε *= 13 200 M^−1^ cm^−1^ at 260 nm). In brief, ctDNA (15 µM) was reacted with ethidium bromide (EtBr, 15 µM). The solution was the excited at 520 nm, and the spectral measurement was measured between 540–750 nm. Thereafter, **C6** was titrated into the solution at a concentration range of 0–337.5 µM. Measurements were carried out in triplicates using 1.0 × 0.4 cm quartz cuvettes. **C6** absorption maxima was monitored at 613 nm, and spectral quenching at 613 nm would indicate the interaction of the C6 with ctDNA due to the displacement of EtBr.

#### 2.4.3 Linear Dichroism

Linear dichroism (LD) experiments were performed by methods that were previously reported [24]. In brief, LD measurements were performed on a Jasco‐1500 magnetic circular dichroism spectropolarimeter, converted for LD, and equipped with an IBM PC and a Jasco J interface. Linear dichroism was defined as

(1) LD(λ) = A// (λ) – A⊥(λ)

*A*// and *A*⊥ are defined as the absorbances of the sample when polarized light was oriented either parallel or perpendicular to the flow direction. The orientation was produced by a Couette cell that rotated at a shear gradient of ±1000 rpm. Each spectrum was accumulated in triplicates from 200 to 600 nm and recorded at 37°C. **C6** concentrations were 50 and 100 µM, and ctDNA concentration used was at 100 µM [31, 32].

#### 2.4.4 In Silico Methods

DNA and protein molecular docking and molecular dynamic simulations were performed by methods previously reported [33, 34]. Full descriptions are reported in the ESI.

## 3 Results and Discussion

### 3.1 Synthesis and Characterization of Ligands and Complexes

**SCHEME 2:**
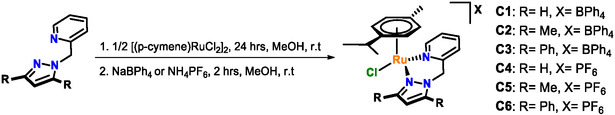
Preparation of pyrazolyl–pyridine Ru(II) metal complexes.

The synthesis and characterization of the Ru(II) pyrazolyl–pyridine complexes described herein have been reported previously in our earlier work and are summarized in Scheme 2 [20]**.** In brief, the complexes were obtained following the established procedure and were characterized by NMR, IR, HRMS, and elemental analysis. In this study, we provide a brief discussion of the synthesis and provide the single‐crystal X‐ray diffraction analysis to further confirm the molecular structures of the complexes. The cationic ruthenium complexes were prepared by the reaction of the synthesized ligands with the dichloro(*p*‐cymene)ruthenium(II) dimer in the presence of methanol for 24 h. This was followed by an anion metathesis reaction using either NaBPh_4_ or NH_4_PF_6_ (Scheme 2), which afforded the complexes as yellow/orange powders (**C1–C6**). The desired complexes were obtained in excellent yields (72–87%) and fully characterized as reported previously [20]**.**

**FIGURE 1:**
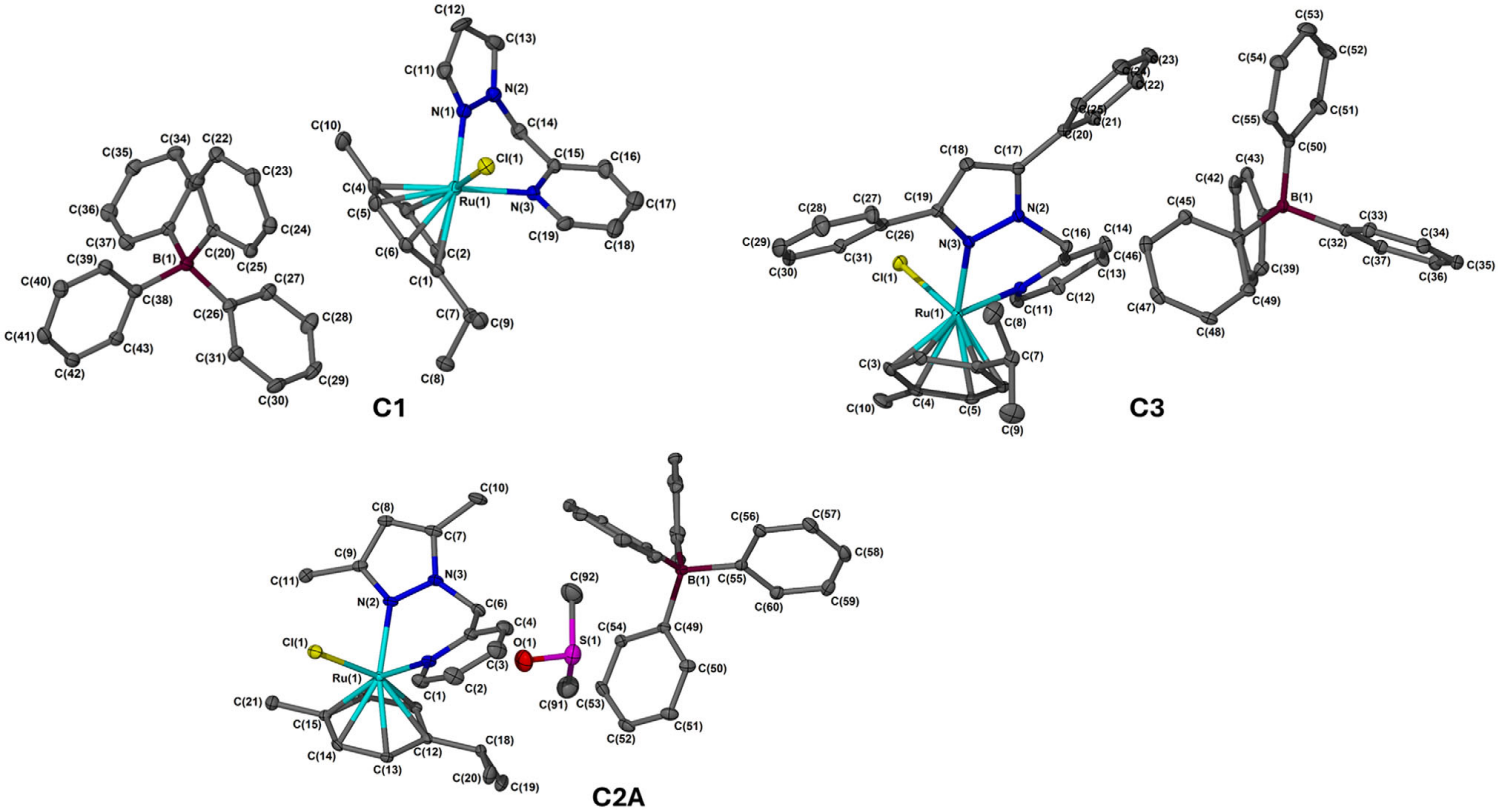
Molecular structures of **C1, C2A,** and **C3** with atomic numbering illustrated with 30% probability ellipsoids. All hydrogen atoms are omitted for clarity.

Single‐crystal X‐ray diffraction analysis provided an unambiguous determination of the molecular structure of complexes **C1–C3.** Notably, **C2** crystallizes with two crystallographically independent molecules in the asymmetric unit, hereafter denoted as **C2A** and **C2B**, which differ slightly in their conformational parameters but share the same coordination environment. Single crystals of **C1** and **C3** were obtained from slow evaporation of a DCM solution, while **C2** was obtained by slow recrystallization in DMSO, as outlined in Figure 1. Crystallographic data is shown in Table S1, while selected bond lengths, bond angles, and torsion angles are shown in Table 1. All the complexes adopt a three‐legged piano‐stool geometry, with the Ru(II) center exhibiting a pseudo‐octahedral coordination environment. The coordination sphere around the ruthenium metal is occupied by a bidentate pyrazole–pyridine ligand, which binds to the ruthenium through the nitrogen atoms of both the pyrazole and pyridine rings, a *p*‐cymene ligand occupying the top face of the stool, and a chloride ligand positioned beneath the metal center. The molecular structures of **C1, C2,** and **C3** display a dissociated ion pair, with the cation and anion significantly separated by distances of 7.167, 8.690, and 6.658 Å (Ru‐B), respectively, aligning with previously reported complexes [35]. The Ru‐N_py_ bond lengths for **C1**, **C2(A)**, **C2(B),** and **C3** are 2.123(3) Å, 2.125(4) Å, 2.127(4) Å, and 2.128(2) Å, respectively, all of which fall within the range found for similar ruthenium complexes and are marginally longer than the corresponding bond to the pyrazole nitrogen (Ru‐N_pz_) [36, 37]. The torsion angles measured were −36.8°, 36.0°, −43.4°, and 35.5°, indicating the non‐planarity of the metallocycles for the complexes. The substituent on the pyrazole ring significantly influenced the spatial arrangement of the anion relative to the cation in the solid state.

**TABLE 1:** Selected bond lengths (Å) and torsion angles (°) of **C1**, **C2**, and **C3**.

Bond Lengths^a^	Torsion Angles	C1	C2(A)	C2(B)	C3
**Ru(1)‐Cl(1)**	—	2.3892(9)	2.4022(11)	—	2.4016(8)
**Ru(1)‐ N(1)**	—	2.096(3)	2.125(4)	—	2.128(2)
**Ru(1)‐N(3)**	—	2.123(3)	—	—	2.120(2)
**Ru(1)‐N(2)**	—	—	2.114(3)	—	—
**Ru(2)‐Cl(2)**	—	—	—	2.4067(10)	—
**Ru(2)‐ N(4)**	—	—	—	2.127(4)	—
**Ru(2)‐N(5)**	—	—	—	2.102(3)	—
—	C(15)‐N(3)‐Ru(1)‐N(1)	−36.8	—	—	—
—	C(5)‐N(1)‐Ru(1)‐N(2)	—	36.0	—	—
—	C(26)‐N(4)‐Ru(2)‐N(5)	—	—	−43.4	—
—	C(15)‐N(1)‐Ru(1)‐N(3)	—	—	—	35.5

a Atoms are labeled as per numbering given in Figure 1.

The BPh_4_^−^ anion was situated below the *p*‐cymene arene ring (**C1**) while the same anion was located above the ligand backbone when methyl (**C2**) and phenyl groups (**C3**) are incorporated onto the pyrazole ligand, respectively.

### 3.2 Antimicrobial Evaluation

The preliminary antimicrobial screening of the small library of Ru complexes was performed at a fixed concentration of 32 µg ml^–1^ of each complex. Growth was assessed after 18 h by comparing it to the untreated control and expressing the result as a percentage. The initial screening included five bacterial species, including Gram‐positive *Staphylococcus aureus* (Sa) (including methicillin‐sensitive and‐resistant (MRSA) strain types), and Gram‐negative strains *Escherichia coli* (Ec), *Klebsiella pneumoniae* (Kp), *Pseudomonas aeruginosa* (Pa), and *Acinetobacter baumannii* (Ab).

The preliminary screen also included two fungi strains, *Candida albicans* (Ca) and *Cryptococcus neoformans* (Cn). Most complexes exhibited some inhibition of the microbial growth rates, as summarized in Table S2, with the exception of **C4** and **C5**, which were therefore not carried over to the minimum inhibition concentration (MIC), cytotoxicity, and hemolytic studies.

From the Ru class of complexes, **C1**‐**C3** were inactive against the majority of the pathogens studied, with MICs over 32 μg mL^−1^ (Table 2). However, there was one exception being **C6**. This is likely due to the fact that **C6** was the most hydrophobic, and this may have resulted in an increase uptake of the complex within the membranes of the bacteria [28, 38]. Among the Gram‐negative bacteria, **C6** exhibited the highest potency against A*. baumannii* (MICs, 16 μg mL^−1^(22 μM), an important nosocomial nonmotile aerobic bacterial pathogen [39]). **C6** had moderate potency against *E. coli* (MICs, 32 μg mL^−1^ (44 µM)) and the gram‐negative *S. aureus* (MICs, 32 μg mL^−1^ (44 μM)) (Table 2). However, unsurprisingly all ruthenium complexes tested had minimal activity toward *P. aeruginosa* (MICs > 32 μg mL^−1^), which is likely due to the poor membrane permeability (only ∼8% that of *E. coli*) and a very effective exogenous regulatory systems, such as the efflux system. This makes *P. aeruginosa* intrinsically resistant to many antibiotics (Table 2) [40].

**TABLE 2:** Inhibitory concentration of the Ru(II) pyrazolyl–pyridine complexes as evaluated against an extended panel of Gram‐positive, Gram‐negative bacteria, fungi and healthy cells.

Gram +			Minimum Inhibition Concentration (MIC), ug ml^−1^			Fungi		HEK−293	RBC
			Gram −						
Compounds	Staphylococcus aureus ATCC 43 300; MRSA	Escherichia coli ATCC 25 922	Klebsiella pneumoniae ATCC 700 603; MDR	Pseudomonas aeruginosa ATCC 27 853	Acinetobacter baumannii ATCC 19 606	Candida albicans ATCC 90 028	Cryptococcus neoformans ATCC 208 821; H99	HK^a^	Hm^b^
**C1**	>32	>32	>32	>32	>32	>32	>32	>32	>32
**C2**	>32	>32	>32	>32	>32	>32	>32	>32	>32
**C3**	>32	>32	>32	>32	>32	>32	>32	>32	>32
**C6**	32	32	>32	>32	16	>32	>32	>32	>32
**Fluconazole**	N.D	N.D	N.D	N.D	N.D	0.125	8	N.D	N.D
**Colistin sulfate**	0.125	1	0.25	0.25	0.25	N.D	N.D	N.D	N.D
**Tamoxifen**	N.D	N.D	N.D	N.D	N.D	N.D	N.D	9	N.D
**Melittin**	N.D	N.D	N.D	N.D	N.D	N.D	N.D	N.D	8.5

a Cytotoxicity (HK) was assessed in HEK‐293 human embryonic kidney cells.

b Haemolytic activity (Hm) was determined using human red blood cells. Values are reported as MIC (µg mL^−1^).

### 3.3 Cytotoxicity (CC50), Hemolytic Activity (HC50), and Cytopathic Effects

To assess the selectivity of the Ru complexes toward microorganisms over mammalian cells, the concentration required to induce 50% cytotoxicity in human embryonic kidney cells (HEK‐293) and 50% hemolysis of human red blood cells (RBCs) were measured for complexes **C1–** **C6** (Table 2). Overall, **C1**‐**C6** exhibited low in vitro cytotoxicity against HEK‐293 and RBC cells (CC_50_, >32 μg mL^−1^ and HC_50_, > 32 μg mL^−1^. This indicates that the Ru complexes are selective for antimicrobial agents. Fluconazole and colistin sulfate were used as positive controls for bacterial pathogens and fungi pathogens, respectively. **C6** exhibited a MIC of 16 µg/mL against *A. baumannii*. Although **C6** exhibited lower potency compared to colistin sulfate, it demonstrated reproducible inhibitory activity against *A. baumannii* within a therapeutically relevant micromolar range (22 μM). Importantly, as a coordination‐based scaffold with a mechanistically distinct mode of action, this complex represents a promising first‐generation framework for further structural optimization aimed at enhancing antibacterial efficacy while potentially mitigating cross‐resistance associated with polymyxins.

### 3.4 Evaluation of Antimicrobial Activity

The rising prevalence of antibiotic resistance to traditional clinical drugs has driven the development of novel and more potent antibiotics, particularly metal‐based antimicrobial agents, over the past decade [41]. Notably, metallodrugs exhibit a tenfold higher hit rate against ESKAPE pathogens compared to purely organic molecules [9]. This has led to the synthesis and investigation of a broad spectrum of organometallic complexes (e.g., Pd [19], Pt [42], Cu [42], Ir [28], and Ru [19, 43, 44]) for their antimicrobial activity. Ru(II) complexes are among the most promising candidates.

Half‐sandwich Ru(II) complexes have been reported to possess moderate to low antimicrobial activity against ESKAPE pathogens, with typical MICs ranging between 0.63 and 2.5 mg mL^−1^ [36, 45, 46, 47]. In this study, the maximum concentration of Ru complex tested was 32 μg mL^−1^, approximately 20‐fold lower than those used in previous studies. From our current library of Ru^II^ complexes, **C6** exhibited the highest activity against *A. baumannii* (MIC: 16 μg mL^−1^).

**C1**–**C5** were inactive against the pathogens tested at the tested concentrated threshold, with MICs > 32 μg mL^−1^ (Table 2), except for **C6**. One possible explanation relates to the counterion: complexes **C1**–**C3** and **C4**–**C6** share the same ligands, but **C1**–**C3** contain BPh_4_^−^ while **C4**–**C6** contain PF_6_^−^. BPh_4_^−^ salts are known to have poor aqueous solubility and tend to aggregate, whereas PF_6_^−^ salts are smaller, more symmetrical, and more uniformly polarizable, which enhances their solubility in aqueous media [48]. Furthermore, BPh_4_^−^ leads to quadrupole ion pairs in DMSO, which prevents catalytic activation and can be inferred here as well. The balance between ion‐pairing clusters and lipophilicity is a competing, contributing factor [49].

Thus, **C1**–**C3** may have been more prone to aggregation under the assay conditions. Interestingly, despite their lower expected solubility, **C1**–**C3** displayed slightly higher activity than **C4** and **C5**. This suggests that increased lipophilicity associated with the BPh_4_^−^ counterion may partially enhance interactions with bacterial membranes, potentially facilitating uptake. However, this effect alone is insufficient to produce significant antimicrobial activity, likely due to competing factors such as aggregation or limited bioavailability. These observations highlight that antimicrobial activity in this series is governed by a delicate balance between lipophilicity and aqueous solubility, rather than by counterion identity alone.

A second factor is the nature of the pyrazole substituent. The phenyl group in **C6** increases overall lipophilicity compared to the hydrogen or methyl groups in **C4** and **C5**, providing a larger aromatic surface area and enhanced π character. This promotes nonpolar interactions, reduces overall polarity, and may improve interactions with hydrophobic domains of biomolecules. Similar trends were observed in a small library of [(η^6^‐p‐cymene)Ru(L)Cl]PF_6_ complexes, where optimal lipophilicity and solubility correlated with maximal antimicrobial activity [50]. The combination of the PF_6_^−^ counterion and the phenyl‐substituted ligand in **C6** likely modulates key properties such as solubility, membrane permeability, and biomolecular interactions [28, 38]. Striking the right balance between lipophilicity, which enhances cell membrane penetration and target interactions, and sufficient solubility in physiological media remains critical for antimicrobial efficacy [51].

**SCHEME 3:**
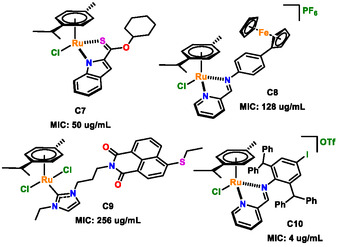
Previously reported ruthenium‐based complexes with antibacterial activity against and *A. baumannii* [52, 53, 54, 55].

Ru(II) arene complexes have emerged as promising candidates in antimicrobial research; however, only a limited number have been systematically evaluated against multidrug‐resistant bacteria (MDRB), particularly *A. baumannii*. Scheme 3 highlights representative arene ruthenium complexes previously reported to exhibit antibacterial activity against *A. baumannii* [52, 53, 54, 55]. **C7–C9** display MIC values at least 3.125‐fold higher than the lead compound identified in this study (**C6**). To date, one of the most active Ru–arene complexes reported is **C10** (Scheme 3), with an MIC of 4 µg mL^−1^, which is approximately fourfold more active than **C6**. Nevertheless, even this most active example remains significantly less potent than the clinical antibiotic colistin sulfate (MIC = 0.25 µg mL^−1^), highlighting the gap between current metal‐based candidates and established last‐resort therapies. Colistin sulfate exhibits potent activity against *A. baumannii* due to its ability to directly disrupt the lipopolysaccharide‐rich outer membrane through a combination of electrostatic and hydrophobic interactions. This membrane‐targeting mechanism enables colistin to bypass the permeability barrier that limits the efficacy of many early‐stage antimicrobial agents. Specifically, colistin binds to lipid A of lipopolysaccharides, displacing stabilizing divalent cations (Ca^2+^ and Mg^2+^), leading to membrane destabilization, increased permeability, and ultimately cell death [56]. However, despite its high potency, colistin is associated with significant clinical limitations, including nephrotoxicity, neurotoxicity, and a narrow therapeutic index [57]. In addition, resistance can arise through lipid A modification, such as the addition of phosphoethanolamine, which reduces colistin binding affinity [58]. These drawbacks emphasize that, while colistin remains highly effective, it is not an ideal long‐term solution and reinforce the need for the development of new antimicrobial agents with improved safety profiles and alternative mechanisms of action. Importantly, this study provides insight into structure–activity relationships that can guide future optimization of this scaffold. Based on the work of Chaudhary et al*.* [55], incorporation of electron‐withdrawing substituents (e.g., halogens) on the pyridine moiety represents a promising strategy to modulate electronic properties and lipophilicity, which may enhance antibacterial activity. However, such effects are highly context‐dependent and likely arise from changes in overall physicochemical properties rather than a simple halogen‐dependent trend. A second avenue for optimization is counterion modification. While PF_6_^−^ is commonly employed, it has been reported to undergo slow hydrolysis under certain conditions, which may affect complex stability [59]. In contrast, triflate (OTf^‐^) offers higher polarity and improved solvation characteristics, which may enhance aqueous stability and influence interactions with biological membranes [60, 61]. Collectively, these findings suggest that fine‐tuning both ligand design and counterion identity will be critical for improving the antimicrobial performance of ruthenium arene complexes against Gram‐negative pathogens such as *A. baumannii*. These results highlight that achieving an optimal balance between lipophilicity, aqueous stability, and membrane permeability will be essential for translating ruthenium‐based complexes into clinically relevant antimicrobials.

### 3.5 Stability of Complex **C6**

**FIGURE 2:**
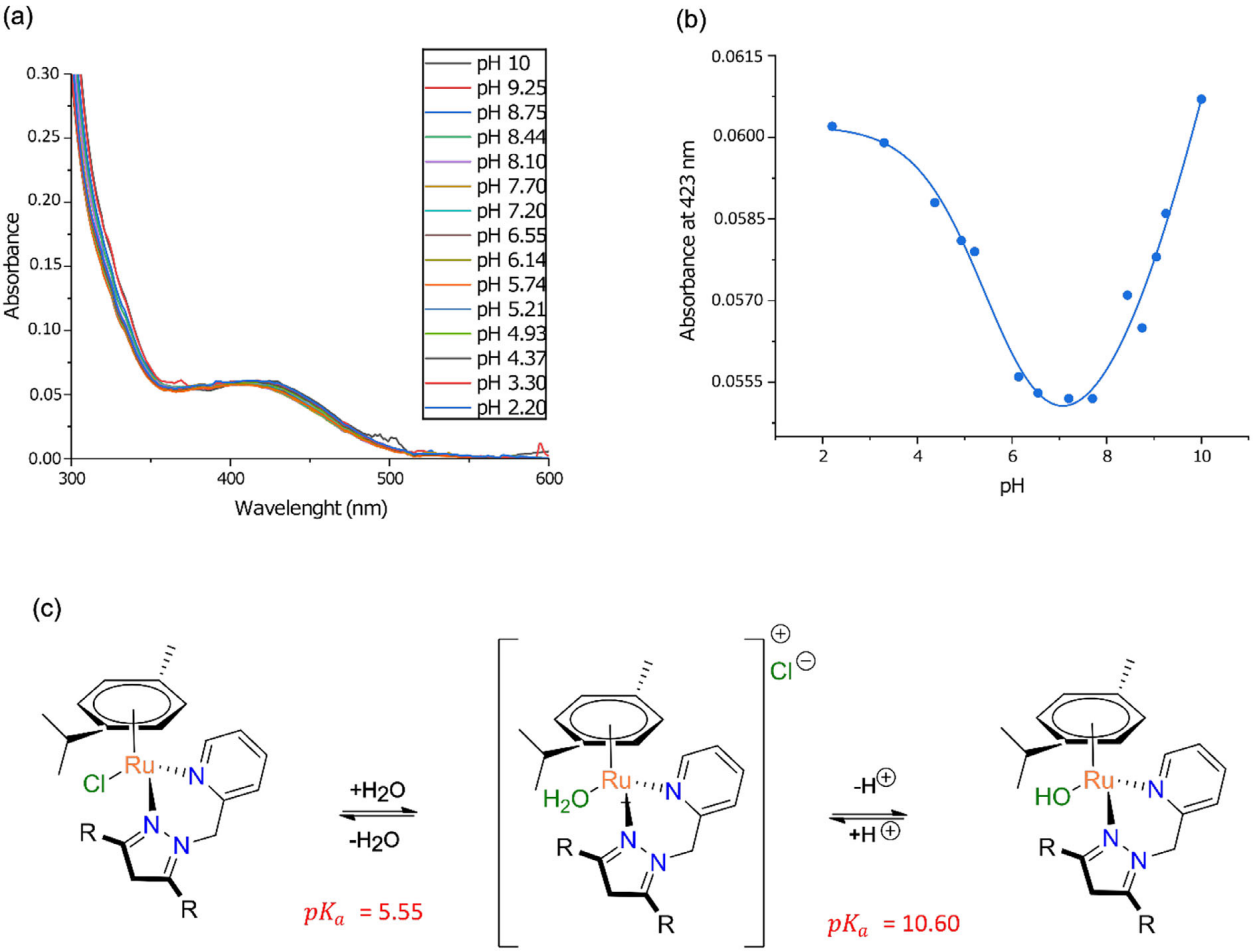
(a) UV‐visible spectra of **C6** recorded as a function of pH at 37°C. (b) Plot of the change in absorbance at 423 nm as a function of pH. The curve is a nonlinear fit of the data to a standard two‐step $pK_{a}$ equilibrium function (AH_2_ ⇌ AH + H^+^, *K*_1_; AH ⇌ A + H^+^, *K*_2_). (c) Proposed scheme summarizing the species distribution gleaned from consideration of the spectra and $pK_{a}$ values delineated from the titration curve. The dominant species at pH 7 involve the equilibrium **C6**‐OH_2_.

The stability of an early stage investigational metallodrug candidate is important for several reasons, such as if it will remain intact in an aqueous environment. Many metallodrug candidates, such as auranofin (Au(I)), and cisplatin (Pt(II)), NAMI‐A (Ru(II)), undergo ligand exchange reactions and ultimately react as a more simple (ionic) metal species both in vitro and in vivo [62, 63]. From our library of ruthenium complexes, mechanistic studies were conducted only on **C6**, as it was the only complex that exhibited antimicrobial activity. The stability and species of **C6** were evaluated through pH speciation studies in a constant ionic strength AMT buffer (5% DMSO) (Figure 2a). The spectral changes of **C6** were evaluated at 423 nm at a pH range of 10–2.2. Upon initial examination, our data revealed a nonlinear bi‐dose response fit, indicative of two distinct $pK_{a}$ values occurring at pH 5.55 (± 0.5) and pH 10.60 (through extrapolation, Figure 2b).

To provide a mechanistic understanding of the observed behavior of **C6** at each $pK_{a}$ value, we present plausible reaction scheme in Figure 2c. The proposed schematic (Figure 2c) indicates that the Cl group on **C6** is replaced by an H_2_O molecule at the pH range where the biophysical assays were conducted (i.e., the aqua species). Therefore, the aqua species of **C6** were used in the following silico methods (vide supra).

### 3.6 Mechanistic Studies

**FIGURE 3:**
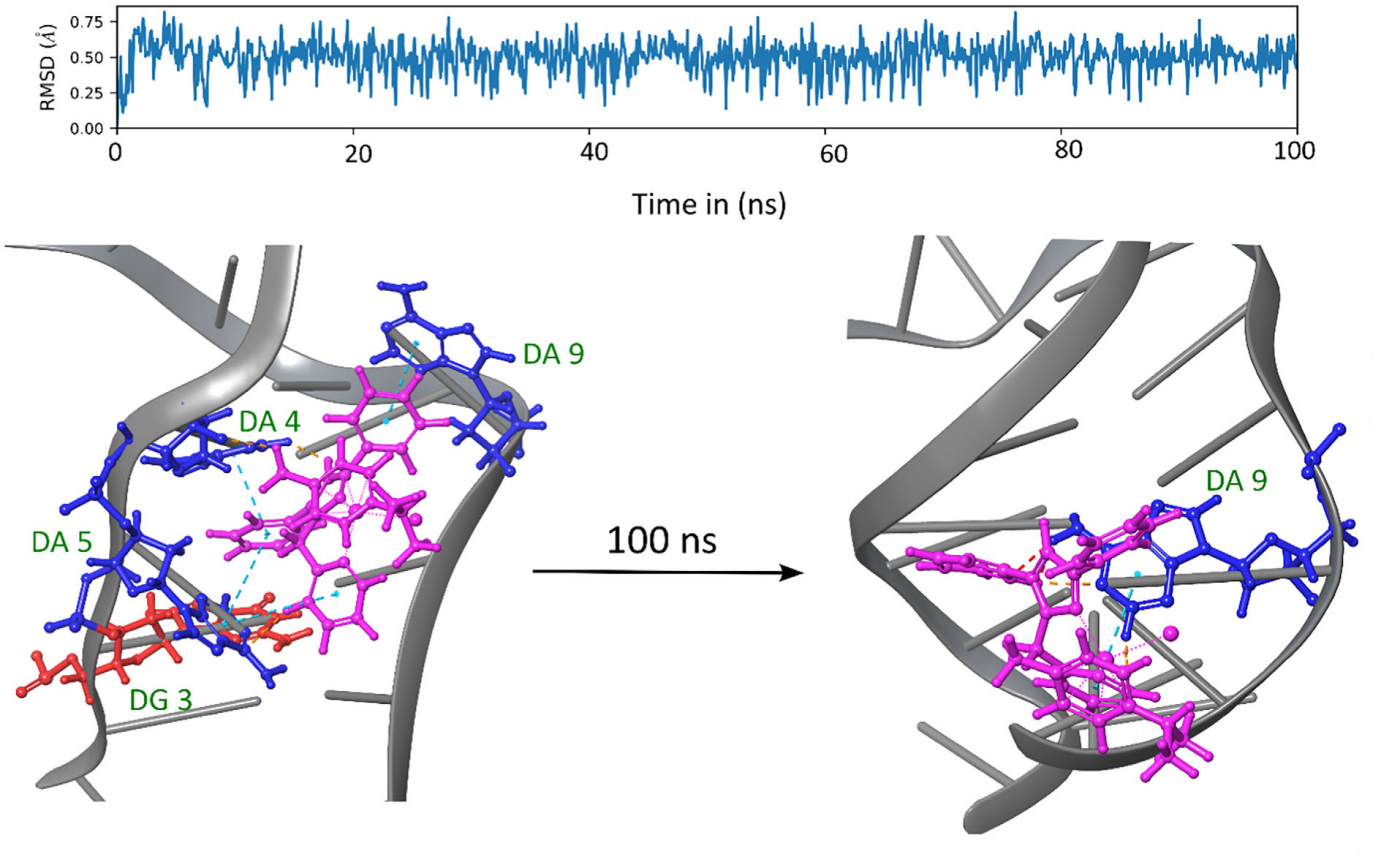
Molecular dynamic simulation (MD) over 100 ns of the best docked GLIDE XP structure of **C6** binding to an oligonucleotide of DNA (PDB 4E1U; 5′‐D(CGGAAATTACCG)‐3′ bound with a cationic ruthenium complex [Ru(bpy)_2_(dppz)]^2+^ [66]. A large target grid was generated for ligand docking at the [Ru(bpy)_2_(dppz)]^2+^ site close to the center of the DNA (with [Ru(bpy)_2_(dppz)]^2+^ removed), spanning 40 × 40 × 40 Å^3^, thereby facilitating a search of alternative binding packets radiating throughout the oligonucleotide. The RMSD indicates that there is minimal fluctuation of **C6** throughout the MD run, however, image docked poses of the fluctuation of **C6** is presented bound within the best XP docked sites. Nucleic acid base pairs are color coded: Adenosine (blue), and Guanosine (red).

To provide preliminary indications of possible target sites to elucidate the potent antimicrobial activity observed for **C6**, we investigated reactions of some active complexes with ctDNA and a number of amino acids. It is well known that Ru^II^ half sandwich complexes are known to interact with DNA [64, 65]; therefore, we assessed whether this could be a mechanism by which **C6** may be functioning. The initial screening was done through silico methods, that is, extra precision docking (XP) and MD simulations (Figure 3).

Hypothetical ligand docking studies were conducted using Glide XP to investigate the potential binding mode of **C6** with DNA. Two DNA X‐ray crystal structures were selected for flexible ligand docking: PDB: 425D (2.80 Å) [67] and PDB: 4E1U (0.92 Å) [66]. It is important to note that ligand docking offers only a predictive model of how a compound might interact with DNA and does not represent a direct simulation of a physical binding event. The primary output of such docking studies is the docking score, which serves as a qualitative indicator and typically does not show quantitative correlation with experimental binding constants or thermodynamic parameters (such as $K_{a}$ or Δ*G* values) [68, 69]. This is because molecular XP docking does not replicate the full complexity of ligand–DNA interactions in solution. As such, docking results are best interpreted qualitatively.

The key observation was that **C6** was predicted to bind to the DNA oligonucleotide, which is consistent with the repetitive nature of AT and GC base pair sequences providing multiple binding sites with comparable affinities. It is worth emphasizing that the DNA structures used for docking are rigid and were crystallized in the presence of either a groove‐binding agent (425D) or an intercalator (4E1U). Therefore, the docking results for C6 were interpreted purely as a likely binding site for the complex and theoretical support for our experimental analysis to follow (vide infra).

Glide XP docking of **C6** to the oligonucleotide indicated a clear preference for the intercalating DNA structure, PDB ID 4E1U (Table 3), as it produced a more favorable Δ*G*_bind_ score as compared to the groove‐binding structure, PDB: 425D. Notably, even the least favorable Δ*G*_bind_ score from 4E1U was lower (i.e., more favorable) than the best pose from 425D. This outcome supports that typical Ru(II) half‐sandwich complexes are known to interact and intercalate with DNA [64, 65]. Additionally, Glide XP docking data indicate that **C6** likely binds and intercalates at the central 5′‐AT‐3′ step of the oligonucleotide (Fig. S1), although the docking score associated with this interaction was relatively modest (ΔG_bind_ = –3.909 kcal mol^−1^). As anticipated, AT‐rich regions were identified as the preferred binding sites, consistent with previous studies showing that these sequences exhibit the lowest binding energies [70].

**TABLE 3:** Summary of GLIDE XP docking scores and selected interaction energy parameters for DNA targets prepared from ligand‐free structures derived from PDB codes 425D (biased towards groove binding), 4E1U (biased towards intercalating). The docking runs were truncated to report only the top‐scoring ligand pose for each ligand. All energies are in units of kcal mol^−1^.

PDB	Binding site residues	XP Gscore	Glide Energy
PDB:425D	AA	−1.412	−54.392
PDB: 4E1U	AG	−3.909	−52.479

Following ligand docking of **C6** to the DNA (PDB: 4E1U), a 100 ns molecular dynamics (MD) simulation (Figure 3) was conducted to assess the stability of the best Glide XP docking pose. MD simulations provide valuable insights into the interactions between macromolecules and ligands over time, capturing the system's dynamic behavior and offering a more accurate reflection of physiological conditions. As shown in Figure 3, the root–mean‐square deviation (RMSD) of **C6** remains relatively stable throughout the simulation trajectory (deviating between 0.25–0.75 Å). At the start of the 100 ns simulation, **C6** is bound to DA(4), DA(5), DA(9) and DG(3). Consequently, throughout the trajectory, **C6** exhibits minimal changes in its binding positions (this can be visualized using MD, as MD force fields allow for more dynamic interactions.

Over the 100 ns, the minimal deviations in the RMSD are attributed to **C6** reorientating into the DNA strand that has now become unwound due to intercalation from the **C6** molecule. This insertion results in the displacement of the stacked base pairs and initiates DNA uncoiling [71]. However, due to this slight conformational change, the **C6** molecule has reorientated itself to form a stable interaction with the minor groove of the oligonucleotide strand. At the end of the 100 ns simulation, the DNA structure undergoes unwinding, disrupting the electrostatic interaction between **C6** and the nucleic acids, and **C6** remains bound to DA(9) only. To postulate a definitive interaction mode of **C6** with DNA, LD spectroscopic experiments were conducted (vide infra).

### 3.7 DNA Binding Studies

**FIGURE 4:**
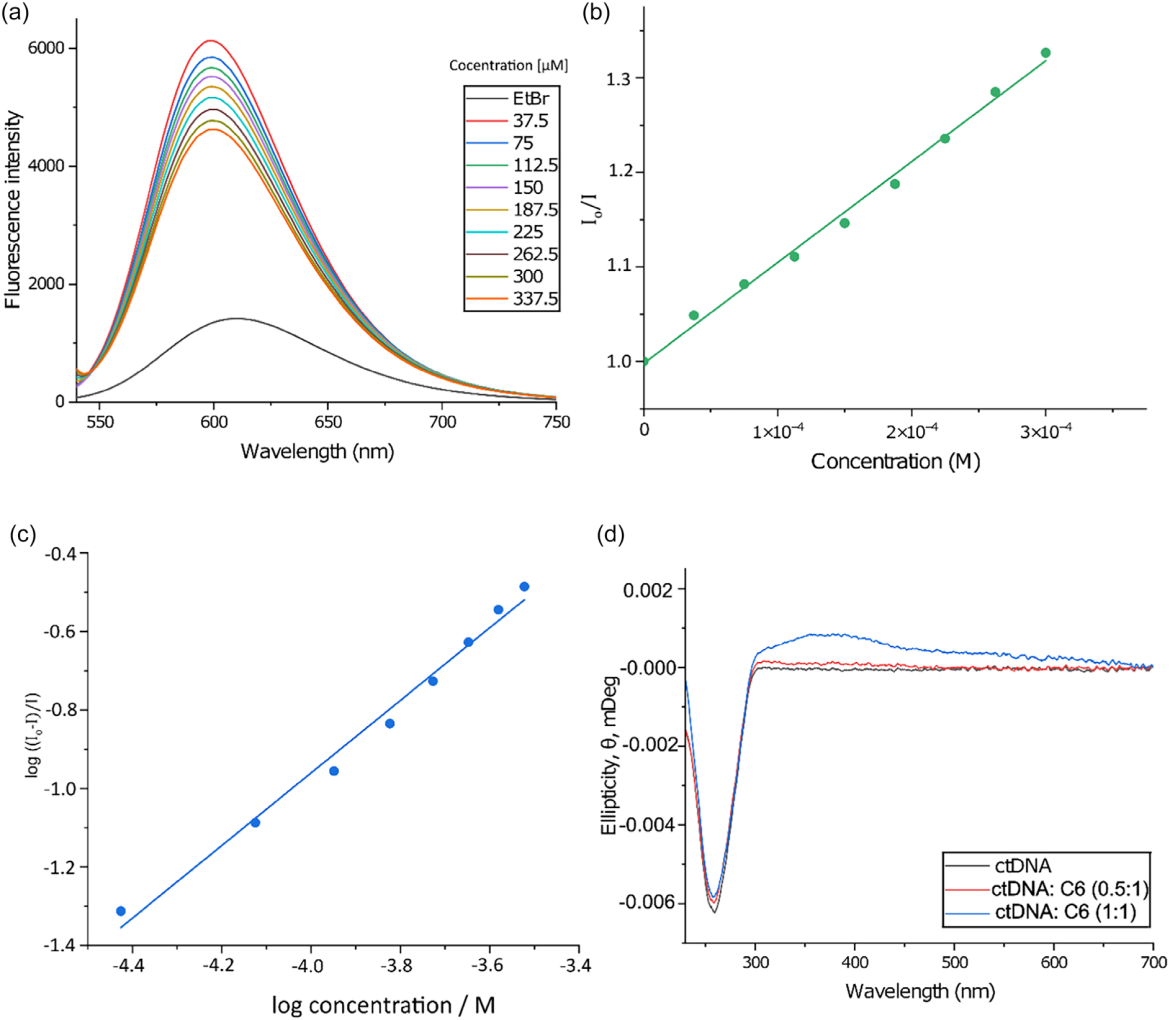
(a) Displacement of intercalated EtBr from ctDNA by **C6** studied by emission spectroscopy (310 K, 50 mM KH_2_PO_4_ buffer; 15 μM ctDNA, 15 μM EtBr). (b) Stern‐Volmer (SV) fluorescence intensity ratio ctDNA•{EtBr} (15.0 μM: 15.0 μM) recorded as a function of the concentration of **C6** in KH_2_PO_4_ (50 mM_4_, pH 7.50). The entire dataset was well‐fitted by Equation (2). (c) Double logarithm plot of the fractional change in fluorescence intensity for ctDNA•{EtBr} (15.0: 15.0 μM) recorded as a function of the concentration of **C6**. The data are described by Equation (3), which affords the affinity constant and stoichiometric coefficient for the reaction. (d) The LD spectrum of **C6** with 100 µM of ctDNA at increasing **C6** concentrations (50 and 100 µM) in KH_2_PO_4_ (10% (v/v) acetonitrile, 50 mM, pH 7.5) at 310 K. The minimum at 260 nm represents ctDNA, and the 300–700 nm broadened band represents an induced **C6** LD spectrum.

To validate the molecular dynamics simulations and assess whether **C6** can interact with DNA as a potential mechanism underlying its antimicrobial activity, we performed a series of DNA binding experiments (Figure 4). Fluorescence spectroscopy was employed using an ethidium bromide (EtBr) displacement assay. The ctDNA•{EtBr} complex and ctDNA•{EtBr} in the presence of **C6** were excited ($\lambda_{520nm}^{Ex}$), and fluorescence emission spectra were recorded over the range of 540–750 nm. Progressive titration of **C6** into the ctDNA•{EtBr} solution resulted in a concentration‐dependent quenching of the extrinsic fluorescence, with an emission maximum observed at ∼613 nm (Figure 4a). The fluorescence intensity decreased monotonically as **C6** concentration increased, indicating competitive displacement of EtBr from the DNA binding sites. These results strongly suggest that **C6** binds in close proximity to EtBr and effectively displaces it from ctDNA, consistent with a dose‐dependent interaction [72, 73].

Fluorescence quenching is normally described by the Stern‐Volmer (SV) equation (Equation (2)) [74],

(2) $$I_{0}/I=1+K_{SV}\left[Q\right]=1+k_{q}\tau_{0}\left[Q\right]$$

where $I_{0}$ is the fluorescence intensity of ctDNA•{EtBr} in this work, and I is the fluorescence intensity of ctDNA•{EtBr} in the presence of **C6**. $K_{SV}$ is the Stern‐Volmer constant (M^−1^), [Q] is the molar concentration of the quencher, $k_{q}$ is the bimolecular quenching rate constant (M^−1^ s^−1^), and $\tau_{0}$ is the average lifetime of ctDNA•{EtBr} fluorescence in the absence of any quencher (23 ns) [75]. The $K_{SV}$, expressing the fluorescence quenching ability of the complexes was found using Equation (2) and was calculated from the slope of the line obtained by plotting $I_{0}/I$ value against [**C6**] values (Figure 4b). The calculated $K_{SV}$ value of **C6** was extrapolated to be 1.067 ± 0.03 × 10^–3^ M^–1^.

Using the fluorescence lifetime $\tau_{0}$ of ctDNA•{EtBr}, the bimolecular quenching constant was calculated as 4.63 × 10^4^ $\;M^{-1}s^{-1}$. This $k_{q}$ is several orders of magnitude below the diffusion‐limited rate (~10^9^–10^10^ M^−1^s^−1^), indicating that collisional (dynamic) quenching is unlikely to be the primary pathway and suggesting a static quenching mechanism (ground–state complex formation) or a microenvironmental protection of the fluorophore. Inner‐filter effects were checked and corrected as described in the methods. Control experiments with free EtBr and buffer only, produced negligible changes in fluorescence.

The measured $K_{SV}$ value for **C6** quenching of ctDNA•{EtBr} was 1.067 × 10^−3^ M^−1^, which is orders of magnitude lower than values reported in many recent studies with metal complexes. For example, Al‐Rashdi et al. (2023) found $K_{SV}$values in the range of ~10^4^–10^5^ M^−1^ when platinum(II) complexes displace EB from DNA; they interpret the quenching as predominantly static, consistent with ground–state complex formation [76]. Similarly, Mvelase et al. (2024) report $K_{SV}$ values of ~10^5^ M^−1^ for Pd‐based complexes, with associated $K_{n}$ in the picosecond regime [77]. Even more recently, Valentová et al. (2024) showed dynamic quenching of ctDNA•{EtBr}, by Cu^II^ complexes, with $K_{SV}$ on the order of 10^3^–10^4^ M^−1^ and very high bimolecular rate constants ($K_{q}$ ~ 10^11^ M^−1^s^−1^), indicating a strong and fast interaction [78].

The measured value of $K_{q}$ of 4.63 × 10^4^ M^−1^ s^−1^ is extremely low compared to diffusion‐limited or static quenching reported in the previously mentioned studies. This suggests a much weaker and potentially a more protected interaction. In summary, the low $K_{SV}$ and $K_{q}$ values in the **C6** system strongly point to a minimal displacement of EtBr, which has an affinity constant of around 3.1 × 10^5^ M [79]. Once again, it needs to be highlighted that these are apparent values extrapolated from an extrinsic fluorescence quenching mechanism.

Typically, the $K_{app}$ values are calculated by utilizing the changes in fluorescence intensities of ctDNA•{EtBr}as a function of complex concentration, where the complexes reduced the fluorescence intensity of ctDNA•{EtBr} solution to 50%. Unfortunately, this could not be performed for the current experiment as ctDNA•{EtBr} was not quenched to 50%, because **C6** had begun to precipitate. Therefore, in order to delineate the $K_{app}$, we used the changes in ctDNA•{EtBr} fluorescence intensity as a function of [**C6**] to calculate the binding equilibrium between complex **C6** and ctDNA. The biophysical parameters that describe the ligand's affinity for the ctDNA ($K_{a}$) and the stoichiometry of the reaction (n) are delineated from a log plot of the emission data as a function of increasing **C6** concentration (Equation (3)) [80],

(3) $$log\left(\frac{I_{0}-I}{I}\right)=logK_{a}+nlog\left[Q\right]$$

where the intercept and slope of the curve give the affinity constant and stoichiometry, respectively. The data has been plotted for **C6** in Figure 4c and was extrapolated to be $logK_{a}$ = 2.73 ± 0.17. It should be noted that we consider the $logK_{a}$ here to be an apparent $logK_{a}$. Unfortunately, we could not calculate the binding affinity of **C6** to ctDNA using UV–vis spectroscopy because the complex undergoes ligand exchange (vide supra) and the electronic spectrum was not consistent. Therefore, the binding constant of **C6** should not be compared to other Ru^II^ half sandwich complexes known to interact with DNA, ranging between $logK_{a}$ 3 – $logK_{a}$ 6.

To confirm the exact binding mode of **C6** to DNA, ultraviolet linear dichroism spectroscopy was employed (UV‐LD). Experiments were conducted in phosphate buffer (10% (v/v) acetonitrile, 50 mM, pH 7.5) using ctDNA and two concentrations of **C6** (50 and 100 µM). The LD spectrum of the ctDNA displays a strong negative signal at 260 nm owing to the absorption of the ctDNA nucleotides (purine and pyrimidine base pairs) [81]. Upon the addition of **C6**, a minimal, concentration‐dependent decrease in the amplitude of the 260 nm minima was observed (Figure 4d). This is typically observed for minor groove binding molecules as they do not substantially affect DNA's rigidity (i.e., DNA stiffening) [31, 82, 83]. A second notable feature was the appearance of an induced dichroic (ICD) signal between 300 and 700 nm, a region where DNA bases do not contribute to absorption; thus, this signal originates exclusively from the **C6** chromophore. The ICD signal also indicates that **C6** is isotropic and cannot be orientated in the flow field. This suggests that **C6** forms a molecular complex with ctDNA, resulting in it becoming orientated, and is a minor groove binder of DNA. The ICD signal positioned above the plane of native ctDNA indicates that **C6** is binding within the minor grove of ctDNA (Figure S2).

While the interaction of **C6** with ctDNA is supported by the biophysical data presented herein, it is important to recognize that such in vitro assays provide only a simplified representation of the intracellular environment. The mechanisms of action of metal‐based antimicrobial agents are often multifaceted, involving interactions with proteins, membranes, and other biomolecules in addition to nucleic acids. Therefore, the observed DNA binding should be interpreted as a potential contributing factor rather than definitive evidence of the primary mechanism of action. Future studies employing more advanced approaches, such as omics‐based analyses, will be necessary to fully elucidate the biological targets and pathways associated with these complexes.

### 3.8 C6 Interaction with Amino Acids

**FIGURE 5:**
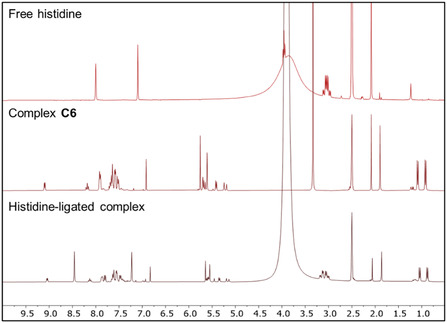
^1^H NMR spectra of free histidine (top), **C6** (middle), and histidine–ligated complex (bottom**)**.

To provide further preliminary indications of possible target sites for **C6**, we investigated reactions of the complex with L–histidine and L–cysteine using ^1^H NMR spectroscopy at pH 7.5 (Figure 5 and **S3**). Samples were prepared and stored at 37°C for 2 h. Analysis via ^1^H NMR spectroscopy revealed no new resonances attributable to L‐cysteine coordination, indicating that under the tested conditions, this amino acid does not bind to **C6**. A parallel experiment was conducted using histidine as a probe. The ^1^H NMR spectrum of free histidine showed distinct aromatic signals at 8.00 and 7.10 ppm, along with aliphatic resonances between 2.08 and 3.05 ppm. Upon mixing histidine with **C6**, several new peaks emerged in the aromatic region, specifically, a sharp signal at 8.46 ppm, a doublet at 7.82 ppm, and a singlet at 7.23 ppm, consistent with coordination of histidine to the complex (Figure 5). In a similar study by Rilak and coworkers, ^1^H NMR spectroscopy was used to investigate the interaction of Ru(II) terpyridine complexes with L‐histidine, where coordination was evidenced by the appearance of new imidazole resonances and significant chemical‐shift changes relative to free histidine. The emergence of these new signals was attributed to histidine binding through the imidazole nitrogen, providing direct NMR evidence for metal–histidine coordination [84].

Many bacterial strains rely on His‐ligated metalloproteins for essential functions such as (i) energy generation through respiratory chain enzymes and (ii) reactive oxygen species detoxification. For example, cytochrome oxidases contain His‐coordinated heme and copper centers, where disruption of histidine coordination would impair electron transfer and respiratory activity. Similarly, superoxide dismutases (SODs) coordinate Cu^2+^, Zn^2+,^ or Fe^2+^ through histidine ligands; displacement or inhibition of this coordination by **C6** could impair SOD activity, weakening bacterial defenses against oxidative stress and promoting cell death. While our preliminary NMR results confirm direct interactions between C6 and histidine, they do not establish whether the complex inhibits His‐ligating proteins in a biological context. To explore this possibility, we performed XP molecular docking against representative His‐ligated enzymes essential for bacterial survival (Table 4).

**TABLE 4:** Glide XP docking of C6 to His‐ligating proteins essential for bacterial survival.

PDB	Protein	XP Gscore	Glide XP	His residue bound to C6
2SOD [85]	Cu/Zn SOD	−1.640	−34.045	His–61
1ISA [86]	Fe SOD	−1.768	−27.607	His–26
1QQW [87]	Catalase	−3.425	−39.600	His–75
1DDZ [88]	Carbonic anhydrase	−1.897	−39.585	His–205
4JGP [89]	Histidine kinase	−3.924	−36.098	His–216

The overall XP binding scores of **C6** to critical His‐ligating proteins were low overall. However, XP docking data is not always a true reflection of true binding data. **C6** bound in a biologically relevant manner to all five Histidine proteins. Even though the docking site was set to 40 × 40 × 40 Å around the active site, the highest binding affinity for **C6** always centered to the protein's active site and coordinated to a His residue. Based on this data, we can propose that **C6** may bind to and inhibit some Histidine containing proteins that are responsible for catalyze activity (Catalase) and regulating ROS (SOD's and Histidine kinases).

The XP docking scores of **C6** with critical His‐ligating proteins were generally low, as well, which is not unexpected given the known limitations of docking in capturing metal–ligand coordination. Nonetheless, **C6** consistently adopted binding poses within the active site regions of all five histidine‐active site containing proteins and coordinated directly to at least one histidine residue. Even with a docking grid of 40 × 40 × 40 Å centered on the catalytic pocket, the most favorable poses were localized to the active sites, suggesting that **C6** may interact in a biologically meaningful way. On this basis, we propose that **C6** has the potential to bind and inhibit histidine‐dependent enzymes involved in catalysis (e.g., catalase) and oxidative stress regulation (e.g., superoxide dismutases, histidine kinases). ^1^H NMR was done with *L*‐cysteine as well; however, this yielded no shift in ^1^H NMR spectrum, (Figure S3).

## 4 Conclusion

We synthesized a library of six Ru(II) pyrazolyl–pyridine complexes (**C1**–**C6**) and evaluated their antimicrobial activity against ESKAPE pathogens. Among these, **C6** emerged as the sole active compound, displaying potent activity with MIC values of 32 µg/ml against *Staphylococcus aureus* ATCC 43 300 (MRSA) and *Escherichia coli* ATCC 25 922, and 16 µg/ml against *Acinetobacter baumannii* ATCC 19 606. The superior activity of C6 is likely related to its increased hydrophobicity relative to **C1**–**C5**, which may enhance cellular uptake by facilitating membrane permeability. Based on our mechanistic investigations, **C6** appears capable of interacting with multiple biological targets. In particular, the compound was found to interact with DNA, potentially through minor groove binding, which may contribute to its antibacterial activity by interfering with key cellular processes such as replication and transcription. However, given the observed affinity of **C6** for histidine, an additional and possibly complementary mode of action may involve binding to histidine‐rich, metal‐dependent proteins, potentially displacing essential cofactors and disrupting enzymatic function. Such interactions could impair bacterial defenses against reactive oxygen species (ROS), ultimately leading to oxidative stress and cell death. Collectively, these findings suggest that the antibacterial activity of **C6** may arise from multiple interacting mechanisms rather than a single dominant target.

## Funding

This study was supported by National Research Foundation (CPRR240312208672, KIC250304301167, KIC250305301301, KIC240827263735, KIC240827263760, 116177, OQM), Wellcome Trust (104797/Z/14/Z).

## Conflicts of Interest

The authors declare no conflicts of interest.

## Supporting information

Supporting Materials

## Data Availability

The data that support the findings of this study are available on request from the corresponding author. The data are not publicly available due to privacy or ethical restrictions.
