# Supplementary material for: Pyrazolyl–Pyridine Ruthenium Complexes: A New Metallic Line of Defense Against Acinetobacter baumannii
Source: Chembiochem. 2026 May 10;27(9):e70371. doi: 10.1002/cbic.70371 (PMC13157889; doi:10.1002/cbic.70371)
Supplement: Supplementary file 1 — Supporting Materials [file CBIC-27-e70371-s001.pdf]

CO-ADD manuscript ESI

Pyrazolyl-Pyridine Ruthenium Complexes: A New Metallic Line of Defense Against *A. baumannii*

M. Cassiem. Joseph, [a,b‡*] Sheldon Sookai,[a‡*] Monika Nowakowska,[a] Rotondwa Mphephu,[a] Angela M. Kavanagh[c], Johannes Zuegg[c], Mark A. T. Blaskovich[c], Andrew J. Swarts [a*]

[a] School of Chemistry, University of the Witwatersrand, Johannesburg, 2050, South Africa.

[b] Department of Chemistry and Polymer Science, Stellenbosch University, Private Bag X1, Stellenbosch, 7599, South Africa.

[c] Centre for Superbug Solutions, Institute for Molecular Bioscience, The University of Queensland, St Lucia QLD 4072 Australia

‡ Equal contribution

1. Experimental Section

1.1 Crystallography

Single crystal X-ray structure determinations were carried out with a four-circle Bruker D8 Venture X-ray diffractometer equipped with a Photon II CPAD area detector and a fine-focus sealed X-ray tube source (Mo anode). Crystals were mounted under Paratone® oil on nylon loops (Hampton Research) and the crystals were kept at 173(1) K during data collection (Oxford CryoStream 700).

1.2 General Spectroscopy

Proton and carbon NMR spectra were recorded on Bruker Avance III 400 and 300 NMR spectrometers at ^{1}H frequencies of 400 MHz and 300 MHz, respectively, and ^{13}C frequencies of 100 MHz and 75 MHz, respectively. Spectra were recorded at 298 K with 5 mm BBOZ or TBIZ probes. Chemical shifts for both proton and carbon were referenced using the solvent signal. MestReNova (version 14.2.1-27684) was used to analyse NMR spectra. FTIR spectra of powder samples were recorded using a Bruker Alpha FTIR spectrometer incorporating a Bruker Platinum® diamond ATR sampling accessory. Spectra were analysed using the OPUS software package on the spectrometer (version 7.5). Mass spectra were recorded with Bruker Compact Q-TOF high-resolution mass spectrometer using Bruker Daltronics HyStar 3.2 SR4 software. Bruker Compass DataAnalysis software (Version 4.3) was used to analyse chromatograms. Samples of pure compounds (typically ca. 10 μg/mL) were prepared in HPLC grade acetonitrile or ethanol for metal chelates and HPLC grade methanol for ligands. Solutions were acidified using 0.1% (V/V) formic acid to obtain spectra in ESI+ mode. Electronic spectra were recorded using either a PerkinElmer Lambda 365 DOUBLE-BEAM spectrometer connected to a Peltier controller and multicell thermostatic cell block or an Analytik Jena Specord210 Plus double-beam instrument fitted with an external water circulating thermostatic bath and thermostatted cell holders. The spectral data were analysed with the spectrometer software or Origin Pro 2020. Spectra were recorded (10-mm pathlength quartz cuvettes) as a function of concentration for both characterization and the determination of molar absorptivity constants.

1.3 Ligand synthesis

A method described by Mphephu et al. was employed [1]. A combination of (2-bromomethyl)pyridine and the produced pyrazoles in toluene, together with 40% aqueous KOH and drops of 40% aqueous tetrabutylammonium bromide, was subjected to reflux for 20 hours. The organic layer was extracted, dried over anhydrous $MgSO_4$, and subsequently evaporated under vacuum. The crude material was rinsed with water and then purified using column chromatography, employing a DCM:Hexane (4:1) eluent mixture to obtain a pure product.

For **L1**, pyrazole was used as the nucleophile. Product: brown oil. Yield: 88%. ^1H NMR (400 MHz, DMSO-d_6) δ(ppm): 8.54 (d, J =4.8 Hz, 1H), 7.86 (d, J =2.4 Hz, 1H), 7.75 (t, J = 15.4 Hz, 1H), 7.49 (d, , J =2.0 Hz, 1H), 7.30 (t, J =11.4 Hz ,1H), 6.96 (d, J = 7.8 Hz, 1H), 6.31 (t, J = 4.0 Hz, 1H), 5.44 (s, CH_2, 2H). $^{13}C\{^1H\}$ NMR (400 MHz, DMSO-d_6) δ(ppm): 157.50, 149.56, 139.73, 137.53, 131.18, 123.17, 121.92, 106.01, 56.95. FTIR (cm^{-1}): Pyridine N=C str (1593.64), pyrazole N=C str 1513.83). ESI-MS (m/z): 160.08 [M^+]

For **L2**, 3,5-dimethyl-1H-pyrazole was used as the nucleophile. Product: yellow oil. Yield: 61%. ^1H NMR (400 MHz, DMSO-d_6) δ(ppm): 8.52 (d, J =7.6 Hz, 1H), 7.74 (t, J =17.0 Hz, 1H), 7.30 (t, J = 12.4 Hz, 1H), 6.85 (d, J =7.8 Hz ,1H), 5.86 (s, 1H), 5.25 (s, CH_2, 2H), 2.18 (s, CH_3, 3H), 2.08 (s, CH_3, 3H). $^{13}C\{^1H\}$ NMR (400 MHz, DMSO-d_6) δ(ppm): 157.78, 149.70, 146.78, 139.75, 137.55, 122.99, 121.57, 105.50, 53.99, 13.79, 11.16. FTIR (cm^{-1}): Pyridine N=C str (1592.80), pyrazole N=C str 1554.10). ESI-MS (m/z): 188.12 [M^+].

For **L3**, 3,5-diphenyl-1H-pyrazole was used as the nucleophile. Product: White solid. Yield: 64%. ^1H NMR (400 MHz, DMSO-d_6) δ(ppm): 8.51 (d, J =3.3 Hz, 1H), 7.83 (d, J =7.0 Hz, 2H), 7.76 (td, J = 17.2 Hz, 1H), 7.58 (d, J =8.0 Hz ,2H), 7.43 (m, 5H), 7.31 (m, 2H), 7.06 (d, J =8.0 Hz, 1H), 6.98 (s, 1H), 5.48 (s, CH_2, 2H). $^{13}C\{^1H\}$ NMR (400 MHz, DMSO-d_6) δ(ppm): 157.38, 150.50, 149.70, 145.87, 137.63, 133.47, 130.42, 129.31, 129.22, 129.13, 128.98, 128.16, 125.63, 125.56, 123.18, 121.95, 104.09, 100.09, 54.97. FTIR (cm^{-1}): Pyridine N=C str (1598.64), pyrazole N=C str 1564.26). ESI-MS (m/z): 312.15 [M^+]. Anal. Calcd for: EA calculated (found): C 81.00(79.54), H 5.50(5.44), N 13.49(13.48).

1.4 Complex Synthesis

A method described by Mphephu et al. was employed for the preparation of Ru(II) complexes [1]. The dichloro(p-cymene)ruthenium(II) dimer was reacted with the pyrazolyl-pyridine ligands in methanol at ambient temperature for 24 hours. Subsequently, the salt was added, and the reaction mixture was stirred for a further two hours. Upon completion of the reaction, the product was separated using filtration (for BPh_4 compounds) and subsequently rinsed with diethyl ether and cold methanol or subjected to recrystallization (for PF_6) using DCM/diethyl ether to get the final product.

Complex **C1** was prepared as outlined in the general procedure, using **L1** and $NaBPh_4$. The product was isolated as a yellow solid. Yield: 87%. ^1H NMR (300 MHz, DMSO-d_6 δ(ppm): 1.20 (dd, J =9 Hz, 6H), 1.93 (s, 3H), 2.80 (m, 1H), 5.34 (dd, J= 15 Hz, 1H), 5.91 (m, J = 9Hz, 2H), 6.04 (m, 3H), 6.59 (t, J =6 Hz, 1H), 6.78 (m, 4H), 6.92 (m, 8H), 7.18 (m, 8H), 7.63 (t, J =15 Hz, 1H), 7.77 (d, J=9 Hz, 1H), 7.84 (d, J =3 Hz, 1H), 8.11 (t, J=9 Hz, 1H), 8.20 (d, J =18 Hz, 1H), 8.95 (d, J = 3Hz, 1H). $^{13}C\{^1H\}$ NMR (400 MHz, DMSO-d_6) δ(ppm): 164.58, 164.09, 163.60, 163.11, 158.03, 155.01, 146.24, 140.77, 136.04, 136.01, 135.47, 126.05, 125.83, 125.81, 125.78, 125.75, 122.01, 108.49, 105.57, 100.60, 86.09, 84.79, 84.50, 83.38, 55.03, 30.75, 22.50, 22.35, 17.93. FTIR (cm^{-1}): pyridine N=C str (1606.92), pyrazole N=C str (1582.71), ESI-MS (m/z): 430.06 [M^+], Anal. Calcd for: EA calculated (found): C 68.94(68.52), H 5.79(5.29), N 5.61(5.57).

Complex **C2** was prepared as outlined in the general procedure, using **L2** and $NaBPh_4$. Product: Yellow solid. Yield: 85%. ^1H NMR (400 MHz, DMSOd_6 δ(ppm): 1.14 (dd, J =24.0 Hz, 6H), 2.06 (s, 3H), 2.46 (d, J =24.0 Hz, CH_3, 6H), 2.79 (m, 1H), 5.10 (d, J= 16 Hz, 1H), 5.76 (d,

J = 16 Hz, 1H), 5.89 (d, J= 8 Hz, 1H), 5.97 (m, 2H), 6.07 (d, J = 10 Hz, 1H), 6.22 (s, 1H), 6.79 (m, 4H), 6.92 (m, 8H), 7.18 (m, 8H), 7.63 (t, J =16 Hz, 1H), 7.95 (d, J = 8 Hz, 1H), 8.09 (t, J= 16 Hz, 1H), 8.92 (d, J =4 Hz, 1H). $^{13}C\{^1H\}$ NMR (400 MHz, DMSO-d_6) δ(ppm): 164.57, 164.08, 163.59, 163.10, 158.16, 155.12, 154.32, 144.04, 140.66, 136.00, 125.93, 125.81, 125.79, 125.75, 125.73, 121.99, 108.62, 106.57, 100.18, 85.37, 84.72, 84.02, 82.74, 51.96, 31.10, 23.07, 21.80, 18.22, 15.65, 11.82 FTIR (cm^{-1}): pyridine N=C str (1603.46), pyrazole N=C str (1579.25), ESI-MS (m/z): 458.09 [M^+], Anal. Calcd for: EA calculated (found): C 69.54(69.02), H 6.10(6.14), N 5.41(5.38).

Complex **C3** was prepared as outlined in the general procedure, using **L3** and $NaBPh_4$. Product: Yellow solid. Yield: 82%. ^1H NMR (300 MHz, DMSO-d_6 δ(ppm): 0.93 (d, J =6.0 Hz, 3H), 1.09 (d, J =6.0 Hz, 3H), 1.89 (s, 3H), 5.18 (d, J =15.0 Hz, 1H), 5.42 (d, J= 6 Hz, 1H), 5.60 (s, 2H), 5.67 (m, 2H), 6.78 (m, 4H), 6.92 (m, 9H), 7.18 (m, 8H), 7.62 (m, 9H), 7.92 (m, 3H), 8.18 (t, J =16 Hz, 1H), 9.11 (d, J = 5.6 Hz, 1H). $^{13}C\{^1H\}$ NMR (400 MHz, DMSO-d_6) δ(ppm): 164.57, 164.08, 163.59, 163.10, 159.67, 159.26, 154.43, 149.69, 148.84, 141.13, 137.64, 136.03, 132.79, 130.64, 129.97, 129.74, 129.55, 129.32, 129.14, 128,98, 128.43, 128.25, 126.05, 125.82, 125.78, 125.76, 125.73, 125.64, 123.19, 121.99, 111.04, 107.19, 100.70, 85.30, 84.55, 83.16, 82.73, 30.95, 22.41, 18.26 FTIR (cm^{-1}): pyridine N=C str (1610.37), pyrazole N=C str (1579.25), ESI-MS (m/z): 582.12 [M^+], Anal. Calcd for: EA calculated (found): C 73.18(73.04), H 5.46(5.48), N 4.74(4.72).

Complex **C4** was prepared as outlined in the general procedure, using **L1** and $AgPF_6$. Product isolated as an orange solid. Yield: 72%. ^1H NMR (400 MHz, DMSO-d_6) δ(ppm): 1.20 (m, 6H), 1.92 (s,3H), 2.81 (m, 1H), 5.35 (d, J= 16 Hz, 1H), 5.91 (m, 2H), 6.03 (m, 2H), 6.59 (s, 1H), 7.63 (t, J= 16, 1H), 7.77 (d, J = 8 Hz, 1H), 7.83 (s, 1H), 8.11 (t, J= 16 Hz, 1H), 8.19 (s, 1H), 8.97 (d, J = 8 Hz, 1H). $^{13}C\{^1H\}$ NMR (400 MHz, DMSO-d_6) δ(ppm): 158.03, 155.03, 146.24, 140.78, 135.46, 126.05, 108.49, 105.58, 100.65, 86.11, 84.82, 84.49, 83.87, 55.03, 30.74, 22.49, 22.33, 17.91. $^{19}F\{^1H\}$ NMR (300 MHz, DMSO-d_6) δ(ppm): -71.08, -69.19. $^{31}P\{^1H\}$ NMR (300 MHz, DMSO-d_6) δ(ppm): -157.37, -131.03. FTIR (cm^{-1}): pyridine N=C str (1603.49), pyrazole N=C str (1575.79), ESI-MS (m/z): 430.06 [M^+], Anal. Calcd for: EA calculated (found): C 39.70(39.58), H 4.03(4.03), N 7.31(7.38).

Complex **C5** was prepared as outlined in the general procedure, using **L2** and NH_4PF_6. Product: Orange solid. Yield: 76.6%. ^1H NMR (400 MHz, DMSO-d_6) δ(ppm): 1.18 (m, 6H), 2.11 (s,3H), 2.49 (m, 2H), 2.54 (m, 4H), 2.83 (m, 1H), 5.14 (d, J= 16 Hz, 1H), 5.82 (d, J= 16 Hz, 1H), 5.95 (d, J= 4 Hz, 1H), 6.02 (d, J= 4 Hz , 2H), 6.10 (d, J= 8 Hz , 1H), 6.21 (s, 1H), 7.66 (t, J= 12, 1H), 8.01 (d, J = 8 Hz, 1H), 8.14 (t, J= 16 Hz, 1H), 8.98 (d, J= 4 Hz, 1H). $^{13}C\{^1H\}$ NMR (400 MHz, DMSO-d_6) δ(ppm): 158.16, 155.13, 154.34, 144.05, 140.66, 125.93, 125,72, 108.62, 106.61, 100.22, 85.37, 84.74, 84.04, 82.77, 51.98, 31.10, 23.04, 21.82, 18.21, 15.64, 11.80. FTIR (cm^{-1}): pyridine N=C str (1603.46), pyrazole N=C str (1561.96), ESI-MS (m/z): 458.09 [M^+], Anal. Calcd for: EA calculated (found): C 41.83(41.87), H 4.51(4.83), N 6.97(6.95).

Complex **C6** was prepared as outlined in the general procedure, using **L3** and NH_4PF_6. Product: Orange solid. Yield: 73%. ^1H NMR (400 MHz, DMSO-d_6) δ(ppm): 0.93 (d, J= 8 Hz 3H), 1.11 (d, J= 4 Hz, 3H), 1.91 (s, 3H), 5.21 (d, J= 16 Hz 1H), 5.25 (d, J= 8 Hz, 1H), 5.65 (s, 2H), 5.72 (m, 2H), 5.80 (s, 3H), 6.94 (s, 1H), 7.55 (d, J = 8 Hz, 2H), 7.61 (s, 3H), 7.66 (m, 4H), 7.94 (d, J = 8 Hz, 3H), 9.12 (d, J = 4 Hz, 1H). $^{13}C\{^1H\}$ NMR (400 MHz, DMSO-d_6) δ(ppm): 159.67, 159.27, 154.45, 148.84, 141.13, 132.11, 130.64, 129.97, 129.74, 129.55, 128.42, 128.25, 126.06, 125.78, 111.03, 107.20, 100.72, 85.30, 84.55, 83.18, 82.75, 55.38, 54.12, 30.95, 22.41, 18.29. FTIR (cm^{-1}): pyridine N=C str (1610.37), pyrazole N=C str (1561.96), ESI-MS (m/z): 582.12 [M^+], Anal. Calcd for: EA calculated (found): C 51.21(51.27), H 4.30(4.72), N 5.78(6.01).

1.5 Biological evaluation

Biological evaluation methods were performed by standardized methods reported by CO-ADD and have been described below:

1.5.1 Antibacterial data collection: Inhibition of bacterial growth was determined by measuring absorbance at 600 nm (OD600), using a Tecan M1000 Pro monochromator plate reader. The percentage of growth inhibition was calculated for each well, using the negative control (medium only) and positive control (bacteria without inhibitors) on the same plate as references.

For all the bacterial assays, each bacterial strain was cultured in Cation-adjusted Mueller-Hinton broth (CAMHB; Bacto Laboratories 212322) at 37°C overnight. A sample of each culture was then diluted 40-fold in fresh CAMHB and incubated at 37°C for 1.5–3 h. The resultant mid-log phase cultures were diluted with CAMHB (CFU mL^{-1} measured by OD_{600}), then added to each well of the compound-containing plates (384-well non-binding surface (NBS) plates; Corning CLS3640), giving a cell density of 5×10^5 CFU mL^{-1} and a total volume of 50 µL (<2% DMSO). Plates were covered and incubated at 37°C for 18 h without shaking. Inhibition of bacterial growth was determined by measuring absorbance at 600 nm (OD_{600}), using media only as negative control and bacteria without inhibitors as positive control. MIC values were determined as the lowest concentration at which the growth was inhibited at ≥ 80% (equivalent to no visible growth by eye). Colistin sulfate (Sigma C4461) and vancomycin HCl (Sigma 861987) were used as internal controls on each plate for Gram-negative and Gram-positive bacteria, respectively. All compounds were tested as 2 independent biological replicates with 2 technical repeats each.

Percentage growth inhibition of an individual sample is calculated based on Negative controls (media only) and Positive Controls (bacterial/fungal media without inhibitors). Please note that negative inhibition values indicate that the growth rate (or OD_{600}) is higher compared to the negative control (bacteria/fungi only, set to 0% inhibition). The growth rates for all bacteria and fungi has a variation of ±10%, which is within the reported normal distribution of bacterial/fungal growth. Any significant variation (or outliers/hits) is identified by the modified *Z*-score, and actives are selected by a combination of inhibition value and *Z*-score analysis. *Z*-score analysis is done to investigate outliers or hits among the samples. The *Z*-score is calculated based on the sample population using a modified *Z*-score method which accounts for possible skewed sample population.

1.5.2 Antifungal data collection: Fungi strains were cultured for 3 days on yeast extract-peptone dextrose (YPD) agar at 30°C. A yeast suspension of 1×10^6 to 5×10^6 CFU mL^{-1} (as determined by OD_{530}) was prepared from five colonies. The suspension was diluted in supplemented YNB and added to each well of the compound containing plates giving a final cell density of fungi suspension of 2.5×10^3 CFU mL^{-1} and 2% DMSO. All plates were covered and incubated at 35°C for 36 h without shaking. Two biological replicates x2 technical replicates were conducted on separate days (final n=4). Growth inhibition of *C. albicans, C. tropicalis and C. glabrata* was determined by measuring absorbance at 630 nm (OD_{630}), while the growth inhibition of *Cryptococcus* spp. and *C. auris* was determined by measuring the difference in absorbance between 600 and 570 nm ($OD_{600\text{-}570}$), after the addition of resazurin (0.01% final concentration) and incubation at 35°C for 2 h. The absorbance was measured using a Biotek Multiflo Synergy HTX plate reader. The percentage of growth inhibition was calculated for each well, using the negative control (media only) and positive control (fungi without inhibitors) on the same plate. The MIC was determined as the lowest concentration at which the growth was fully inhibited, defined by an inhibition ≥80%. In addition, the maximal percentage of growth inhibition is reported as D_{Max}, indicating any compounds with marginal activity.

1.5.3 Cytotoxicity data collection: HEK-293 ATCC CRL-1573 human embryonic kidney cells were counted manually in a Neubauer haemocytometer and added to compound-containing plates (384- well plates, tissue culture treated (TC); Corning CLS3712) giving a final density of 5000

cells/well and a total volume of 50 µL (<0.5% DMSO), using Dulbecco's modified Eagle's medium (DMEM; Life Technologies 11995–073) with 10% foetal bovine serum (FBS; GE SH30084.03). The cells were incubated together with the compounds for 20 h at 37°C in 5% CO_2. Cytotoxicity (or cell viability) was measured by fluorescence, λ^{ex}=560/10 nm, λ^{em}=590/10 nm (F560/590), after addition of 5 µL of 25 µg mL^{-1} resazurin (2.3 µg mL^{-1} final concentration; Sigma R7017) and after further incubation for 3 h at 37°C in 5% CO_2, using media only as negative control and cells without inhibitors as positive control. CC50 (concentration at 50% cytotoxicity) were calculated by curve fitting the inhibition values vs. log(concentration) using a sigmoidal dose response function, with variable fitting values for bottom, top and slope. Tamoxifen (Sigma T5648) was used as internal control on each plate.

Growth inhibition of HEK293 cells was determined measuring fluorescence at λ^{ex}=530/10 nm and λ^{em}=590/10 nm (F560/590), after the addition of resazurin (25 µg mL^{-1} final concentration) and incubation at 37°C and 5% CO_2, for an additional 3 h. The fluorescence was measured using a Tecan M1000 Pro monochromator plate reader. The percentage of growth inhibition was calculated for each well, using the negative control (medium only) and positive control (cell culture without inhibitors) on the same plate as references. Concentration at 50% cytotoxicity (CC50) values were calculated by fitting the curve of the inhibition values vs. log(concentration) using Sigmoidal dose-response function, with variable values for bottom, top and slope. The curve fitting is implemented using Pipeline Pilot's dose-response component (giving similar results to similar tools such as Origins). Any value with > indicates a sample with no activity (low D_{Max} value) or samples with CC50 values above the maximum tested concentration (higher D_{Max} value).

1.5.4 Haemolysis data collection: Human whole blood (Australian Red Cross) was washed three times with 3 volumes of 0.9% NaCl and resuspended in a concentration of 0.5×108 cells mL^{-1}, determined by manual cell count in a Neubauer haemocytometer. Washed cells were added to compound-containing plates (384-well polypropylene plates (PP); Corning 3657) for a final volume of 50 µL, shaken and incubated for 1 h at 37°C. After incubation, the plates were centrifuged at 1000*g* for 10 min to pellet cells and debris, 25 µL of the supernatant was then transferred to reading plates (384-well, polystyrene plated (PS), Corning CLS3680), with haemolysis determined by measuring the supernatant absorbance at 405 nm (OD_{405}), using cells without inhibitors as negative control and cells with 1% Triton X-100 (Sigma T8787) as positive control. HC10 and HC50 (concentration at 10% and 50% haemolysis, respectively) were calculated by curve fitting the inhibition values vs. log(concentration) using a sigmoidal dose-response function with variable fitting values for top, bottom and slope. Melittin (Sigma M2272) was used as an internal control on each plate. The use of human blood (sourced from the Australian Red Cross Blood Service) for haemolysis assays was approved by the University of Queensland Institutional Human Research Ethics Committee, Approval Number 2014000031.

Concentration at 10% haemolytic activity (HC10) values were calculated by fitting the curve of the inhibition values vs. log(concentration) using Sigmoidal dose-response function, with variable values for bottom, top and slope. The curve fitting is implemented using Pipeline Pilot's dose-response component (giving similar results to similar tools such as Origin). The curve fitting resulted in HC50 (50%) values, which are converted into HC10 by HC10=HC50×(10/90)(1/slope); Any value with > indicates a sample with no activity (low D_{Max} value) or samples with HC10 values above the maximum tested concentration (higher D_{Max} value).

1.6 *In silico* methods

DNA and proteins molecular docking and molecular dynamic simulations were performed by methods previously reported [1,2].

Detailed methodologies are described below.

1.6.1 Ligand preparation: The ruthenium metal complex used for docking was first optimized with DFT at the CAM-B3LYP/LANL2DZ level of theory to determine the likely coordination geometry at the metal centre and ligand conformation. The optimized DFT structures were then prepared for using the OPLS2005 force field with standard parameters for LigPrep (LigPrep, Schrödinger, LLC, New York, NY, USA, 2021) to generate files associated with Schrödinger. The force field used in this study did not include parameters for the metal chelates under investigation. As a result, zero-order bonds were applied between the metal ion and the organic ligand framework. This approach allows the force field to handle the metal-ligand interactions through parameterized electrostatic forces, disregarding the covalent nature of these bonds. This simplification is necessary for docking metal complexes using Schrödinger software.

1.6.2 DNA preparation: Two DNA structures PDB:425D (2.80 Å)[3] and PDB: 4E1U (0.92 Å)[4]were retrieved for preparation using the Protein Preparation Wizard [5] within the Schrödinger Suite 2022–4. The structures contain co- crystallised water molecules and/or ligands. The DNA was selected, inverted, and all other components of the crystal were deleted, leaving only a single DNA macromolecule. All DNA structures were pre-processed at pH 7.4 using Epik [6] to generate heteroatom ionization states. The missing terminal phosphate backbones were then added in Maestro. Hydrogen-bond assignment was optimized at pH 7.4, followed by restrained minimization using the OPLS2005[7] force field, with heavy atoms converging to a root-mean-square deviation (RMSD) of 0.30 Å. PDB:425D is well-suited for generating groove-binding poses, while PDB:4E1U is ideal for generating intercalated docking poses.

1.6.3 Ligand docking: The prepared ruthenium metal complex was docked into the DNA using Glide[8] to identify potential binding sites for the metal chelate on DNA. The receptor grid was centred on nucleobases near the centre of the DNA strand, with dimensions of $40 \times 40 \times 40$ Å^3, ensuring that the entire DNA was sampled for potential binding sites. Extra precision (XP) [9] docking was then employed to generate binding poses on the DNA.

1.6.4 Molecular dynamics: Molecular dynamics (MD) simulations were conducted on the best-docked DNA-ligand complexes using Desmond [10] and the OPLS2005 force field. Prior to simulation, the DNA-ligand complexes were pre-processed, and the System Builder in Desmond was used to solvate the system with TIP3P [11] water molecules. The biomolecular system was placed in an orthorhombic box with a 5 Å buffer between the box boundary and the DNA-ligand complex, then neutralized with Na^+ as needed. The simulations were run for 100 ns, with approximately 1000 frames recorded at 100 ps intervals. Before starting the simulation, the system was relaxed and equilibrated under the NPT ensemble[12] at 310 K and 1.01 bar. The resulting trajectories were analyzed using Maestro.

1.6.5 XP Protein docking experiments: Five proteins that contain Histidine in their actives sites Cu/Zn SOD (2SOD [13]), Fe SOD (1ISA [14]), Catalyse (1QQW [15]), Carbonic anhydrase (1DDZ [16]) and Histidine Kinase (4JGP [17]) were prepared using pre-processed. The proteins active sites were selected as the centroids of the grid box with a grid size of $40 \times 40 \times 40$ Å. The binding of **C6** was directed to histidine by imposing positional constraints during grid generation. The XP docking was then performed with free rotation allowed for the selected residues and the grid-based constraint was applied. The feature definition for the constraint was applied as the SMARTS pattern for Ru(II), [Ru+2]. This constrained docking restricts the ruthenium centre of the complex to within 1.8 and 3.3 Å of the histidine residues.

2. Supplementary Figures

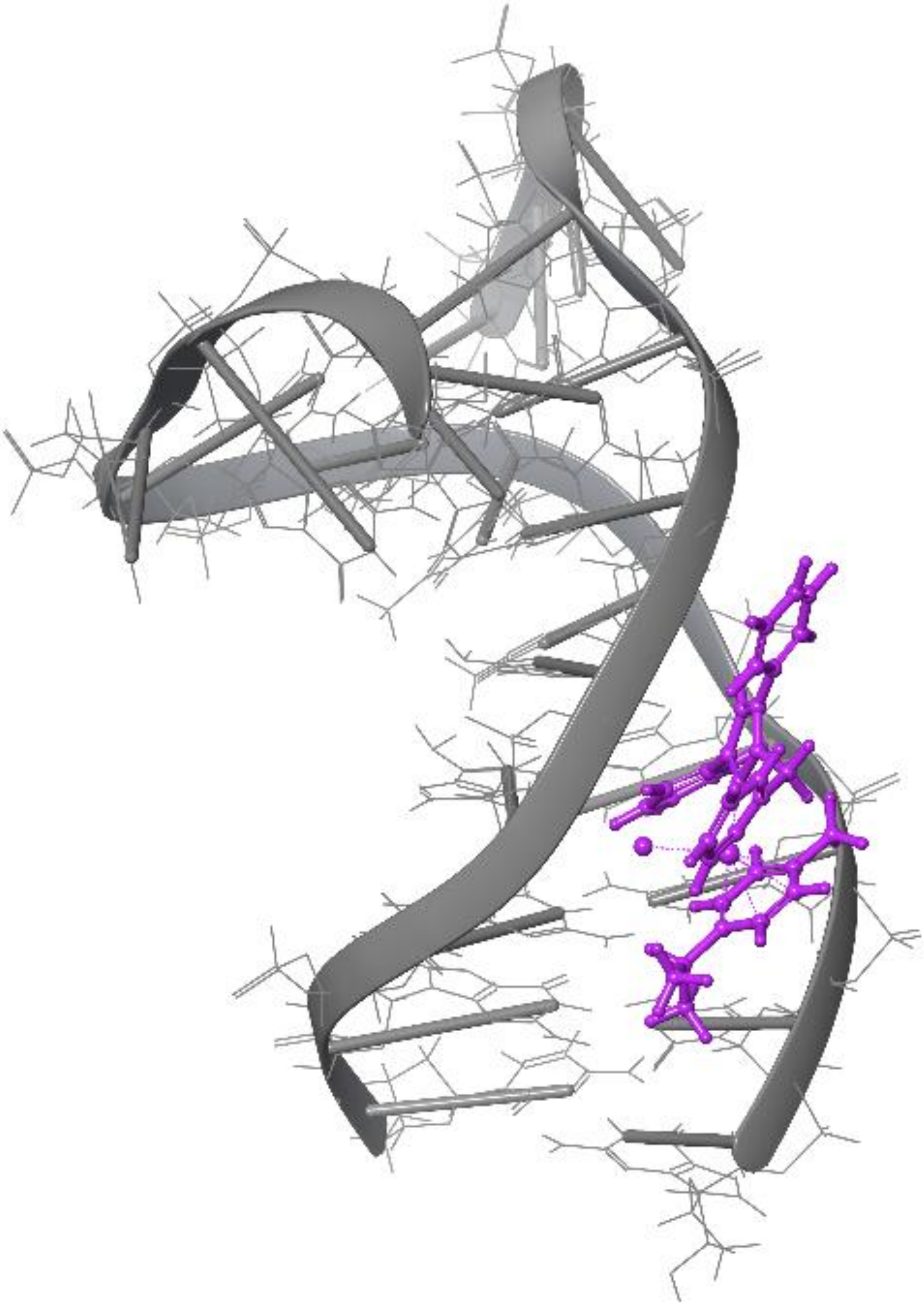

Figure S1. Glide XP docking data indicate that **C6** likely binds and intercalates at the central 5′-AT-3′ step of the oligonucleotide

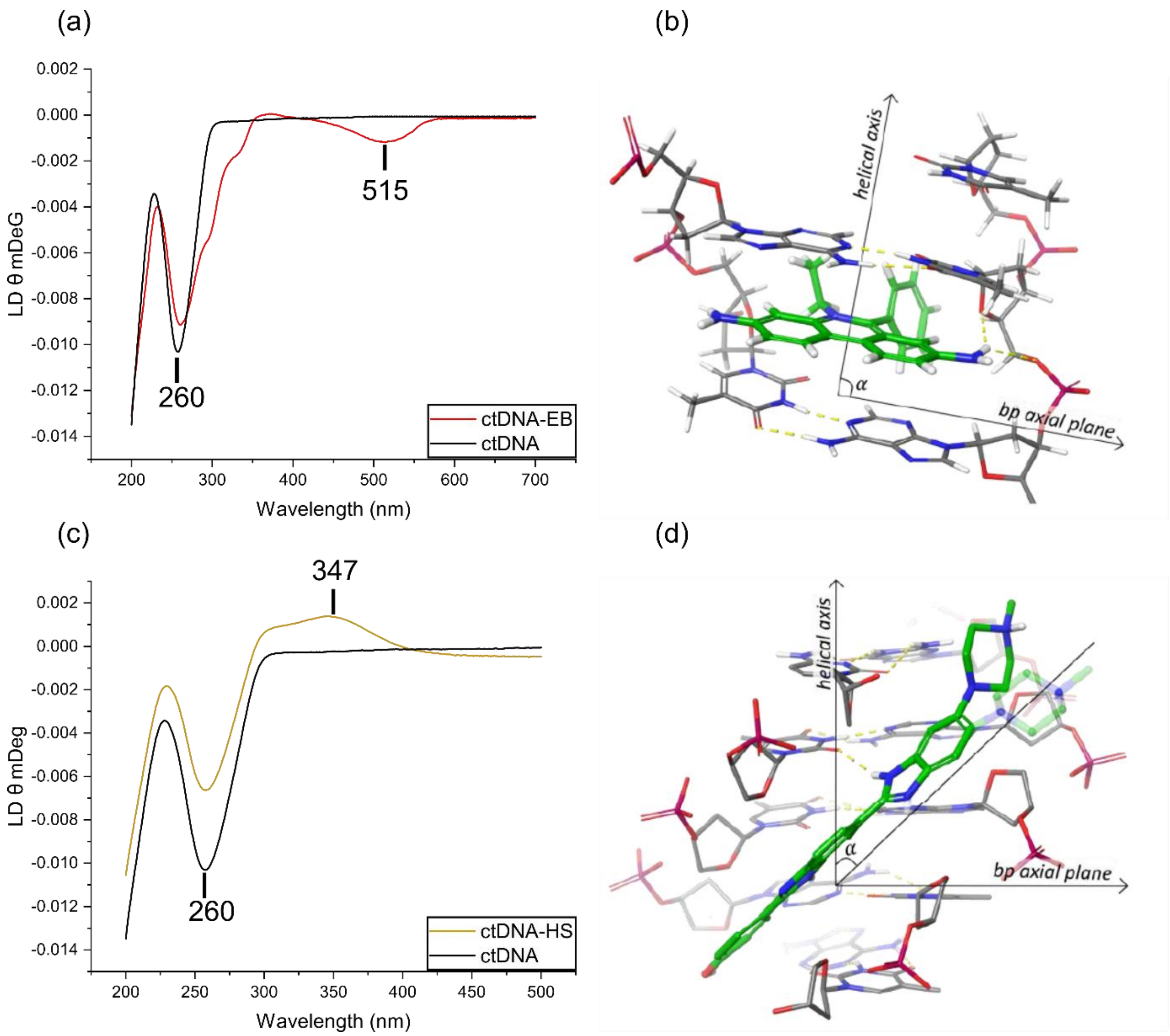

Figure S2 (a) The LD spectrum of EB with ctDNA, with an induced LD at 515 nm indicating binding of EB to ctDNA. (b) The best docking pose generated of EB intercalated with double-stranded DNA (PDB: 2O1I).[18] (c) The LD spectrum of HS with ctDNA, with an induced LD at 347 nm indicating binding of HS to ctDNA. (d) An XRD crystal structure of HS in the minor groove of DNA (PDB: 8BNA).[19]

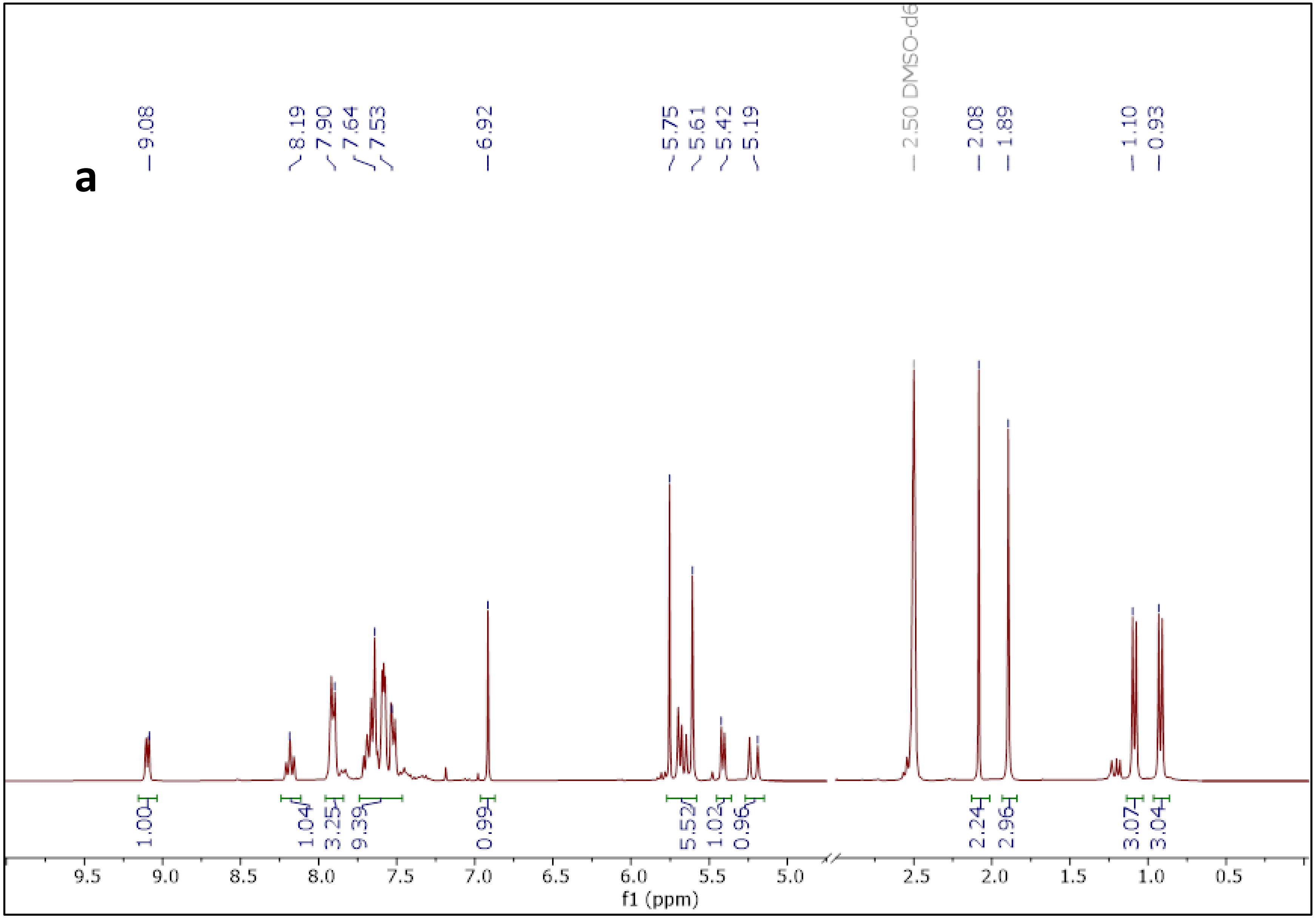
a
9.08
8.19
7.90
7.64
7.53
6.92
5.75
5.61
5.42
5.19
2.50 DMSO-d6
2.08
1.89
1.10
0.93
1.00
1.04
3.25
9.39
0.99
5.52
1.02
0.96
2.24
2.96
3.07
3.04
9.5
9.0
8.5
8.0
7.5
7.0
6.5
6.0
5.5
5.0
2.5
2.0
1.5
1.0
0.5
f1 (ppm)

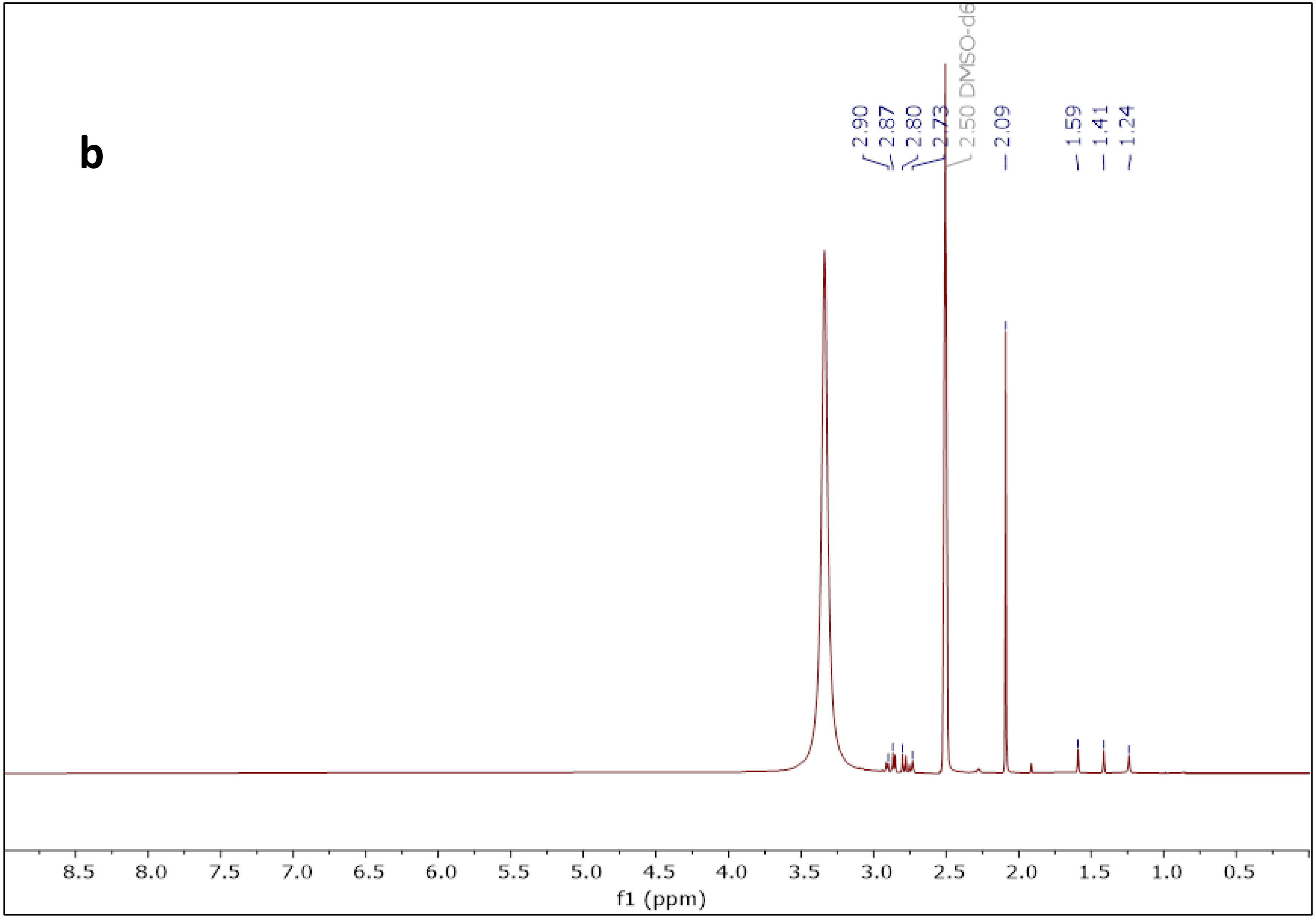
b
2.90
2.87
2.80
2.73
2.50 DMSO-d6
2.09
1.59
1.41
1.24
8.5
8.0
7.5
7.0
6.5
6.0
5.5
5.0
4.5
4.0
3.5
3.0
2.5
2.0
1.5
1.0
0.5
f1 (ppm)

c

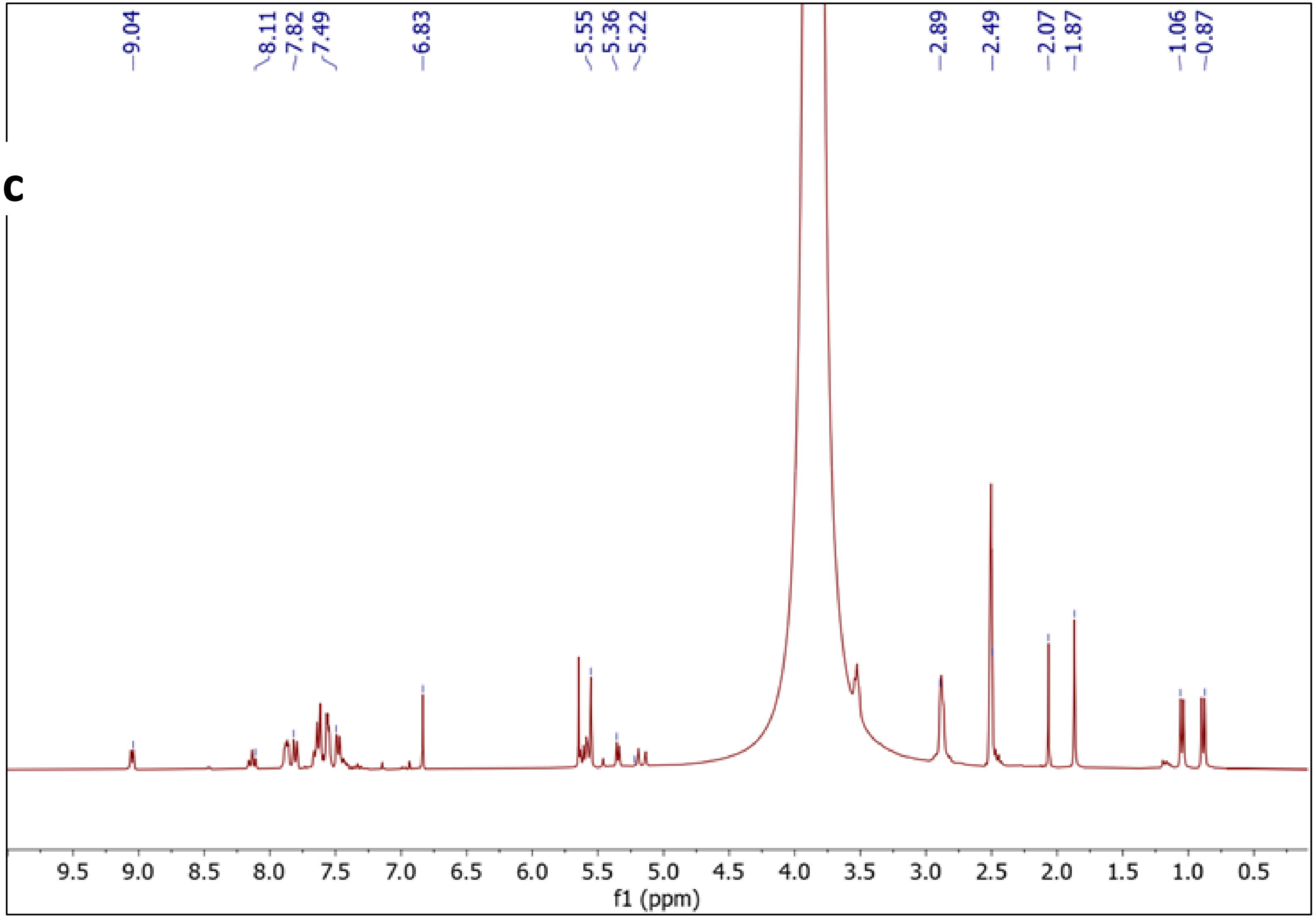

Figure S3 (a) ^{1}H NMR spectrum for **C6**, recorded in DMSO-d_6. (b ^{1}H NMR of L-Cysteine, recorded in DMSO-d_6. (c) ^{1}H NMR of L-Cysteine with **C6**, recorded in DMSO-d_6.

Table S1: Crystallographic data and structure refinement parameters of ruthenium complexes **C1**, **C2**, and **C3**.

**Parameters**	**C1**	**C2**	**C3**
**Empirical formula**	$C_{43}H_{43}BClN_3Ru$	$C_{92}H_{100}B_2Cl_2N_3ORu_2S$	$C_{56}H_{57}BClN_3Ru$
**Mr (g/mol)**	749.13	1632.49	901.16
**Crystal system**	Monoclinic	Triclinic	Monoclinic
**Space group**	$P2_1/c$	P-1	$P2_1/n$
a **(Å)**	12.2464(6)	9.8989(8)	10.6877(7)
b **(Å)**	14.1607(7)	18.3121(2)	34.945(2)
c **(Å)**	21.0438(9)	23.124(2)	12.9323(8)
α **(deg)**	90	80.471(4)	90
β **(deg)**	102.042(2)	80.065(4)	94.161(2)
γ **(deg)**	90	75.591(6)	90
**Crystal size (mm3)**	0.212 x 0.093 x 0.041	0.293 x 0.068 x 0.053	0.185 x 0.109 x 0.102
**Volume (Å3)**	3569.1(3)	3965.7(6)	4817.2(5)
**Z**	4	2	4
**Dcalc (g/cm3)**	1.394	1.367	1.312
**F(000)**	1552.0	1700.0	1984.0
**λ (MoK*a*) (Å)**	0.71073	0.71073	0.71073
**Temperature (K)**	173.00	173.00	173.00
**2*θ* min (deg)**	3.4	2.316	3.366
**2*θ* max (deg)**	55.998	56.624	56.648
**μ (mm-1)**	0.550	0.527	0.426
**Goodness-of-fit on F2**	1.034	1.104	1.203
**Final *R*1 indices [*I*>2σ(*I*)]**	0.0474	0.0628	0.0600
wR2 **(all reflections)**	0.1135	0.1485	0.1154
**Flack x parametes**	-	-	-

Table S2: The results from the preliminary screening of the Ru(II) pyrazolyl–pyridine complexes at a single concentration against a selection of bacteria and fungi.

			Percentage growth inhibition				
	Gram +			Gram -		Fungi	
Compounds	Staphylococcus aureus ATCC 43300; MRSA[a]	Escherichia coli ATCC 25922[a]	Klebsiella pneumoniae ATCC 700603; MDR[a]	Pseudomonas aeruginosa ATCC 27853[a]	Acinetobacter baumannii ATCC 19606[a]	Candida albicans ATCC 90028[a]	Cryptococcus neoformans ATCC 208821; H99[a]
**C1**	14.63 (1.8)	-23.7 (0.6)	-7.21 (0.8)	-1.8 (1.4)	-3.62 (8.2)	91.85 (11)	65.94(10)
**C2**	1.46 (0.22)	-39.06 (0.8)	-19.56 (4)	-21.27 (3)	-17.79 (3)	95.93(2)	9.96 (7)
**C3**	2.94 (7.8)	-224.43 (2.4)	-4.46 (2)	-14.83 (5)	-2.97 (0.1)	48.54 (46)	-27.91 (1)
**C4**	7.27 (3)	-8.2 (1.4)	6.13 (5)	-15.38 (2)	6.23 (0.7)	-1.88 (2)	-19.80(8)
**C5**	-15.43 (6.4)	-4.02 (1.8)	4,31 (0.8)	-8.47 (3)	2.595	-2.10 (6.4)	-50.40 (8)
**C6**	0.71 (4.2)	0.73 (1.8)	9.47 (2)	14.94 (2)	14.79 (3)	5.92 (3)	-11.2(14)

[a]Paranthesis indicates standard deviation of replicate results.

3. References

1. Bracken, M.L.; Moyo-Gwete, T.; Sookai, S.; Ayres, F.; Munro, O.Q. Copper Pincer Targets SARS-CoV-2 Omicron, Delta, and Wild-Type Receptor Binding Domains. *Inorganic Chemistry Communications* **2025**, *179*, 114887, doi:10.1016/j.inoche.2025.114887.
2. Sookai, S.; Munro, O.Q. Delineating the Binding Site of a Series of Gold(III) Schiff Base Chelates on HSA *via* Experimentation and *in Silico* Methods. *International Journal of Biological Macromolecules* **2025**, *315*, 144317, doi:10.1016/j.ijbiomac.2025.144317.
3. Rozenberg, H.; Rabinovich, D.; Frolow, F.; Hegde, R.S.; Shakked, Z. Structural Code for DNA Recognition Revealed in Crystal Structures of Papillomavirus E2-DNA Targets. *Proc. Natl. Acad. Sci. U.S.A.* **1998**, *95*, 15194–15199, doi:10.1073/pnas.95.26.15194.
4. Song, H.; Kaiser, J.T.; Barton, J.K. Crystal Structure of Δ-[Ru (Bpy) 2 Dppz] 2+ Bound to Mismatched DNA Reveals Side-by-Side Metalloinsertion and Intercalation. *Nat. Chem.* **2012**, *4*, 615–620.
5. Madhavi Sastry, G.; Adzhigirey, M.; Day, T.; Annabhimoju, R.; Sherman, W. Protein and Ligand Preparation: Parameters, Protocols, and Influence on Virtual Screening Enrichments. *J Comput Aided Mol Des* **2013**, *27*, 221–234, doi:10.1007/s10822-013-9644-8.
6. Shelley, J.C.; Cholleti, A.; Frye, L.L.; Greenwood, J.R.; Timlin, M.R.; Uchimaya, M. Epik: A Software Program for pK a Prediction and Protonation State Generation for Drug-like Molecules. *J. Comput. Aided. Mol. Des.* **2007**, *21*, 681–691.
7. Banks, J.L.; Beard, H.S.; Cao, Y.; Cho, A.E.; Damm, W.; Farid, R.; Felts, A.K.; Halgren, T.A.; Mainz, D.T.; Maple, J.R.; et al. Integrated Modeling Program, Applied Chemical Theory (IMPACT). *J Comput Chem* **2005**, *26*, 1752–1780, doi:10.1002/jcc.20292.
8. Friesner, R.A.; Banks, J.L.; Murphy, R.B.; Halgren, T.A.; Klicic, J.J.; Mainz, D.T.; Repasky, M.P.; Knoll, E.H.; Shelley, M.; Perry, J.K.; et al. Glide: A New Approach for Rapid, Accurate Docking and Scoring. 1. Method and Assessment of Docking Accuracy. *J. Med. Chem.* **2004**, *47*, 1739–1749, doi:10.1021/jm0306430.
9. Friesner, R.A.; Murphy, R.B.; Repasky, M.P.; Frye, L.L.; Greenwood, J.R.; Halgren, T.A.; Sanschagrin, P.C.; Mainz, D.T. Extra Precision Glide: Docking and Scoring Incorporating a Model of Hydrophobic Enclosure for Protein−Ligand Complexes. *J. Med. Chem.* **2006**, *49*, 6177–6196, doi:10.1021/jm051256o.
10. Bowers, K.J.; Sacerdoti, F.D.; Salmon, J.K.; Shan, Y.; Shaw, D.E.; Chow, E.; Xu, H.; Dror, R.O.; Eastwood, M.P.; Gregersen, B.A.; et al. Molecular Dynamics---Scalable Algorithms for Molecular Dynamics Simulations on Commodity Clusters. In Proceedings of the Proceedings of the 2006 ACM/IEEE conference on Supercomputing - SC ’06; ACM Press: Tampa, Florida, 2006; p. 84.
11. Jorgensen, W.L.; Chandrasekhar, J.; Madura, J.D.; Impey, R.W.; Klein, M.L. Comparison of Simple Potential Functions for Simulating Liquid Water. *The Journal of chemical physics* **1983**, *79*, 926–935.
12. Jorgensen, W.L. Convergence of Monte Carlo Simulations of Liquid Water in the NPT Ensemble. *Chemical Physics Letters* **1982**, *92*, 405–410.
13. Tainer, J.A.; Getzoff, E.D.; Beem, K.M.; Richardson, J.S.; Richardson, D.C. Determination and Analysis of the 2 Å Structure of Copper, Zinc Superoxide Dismutase. *Journal of Molecular Biology* **1982**, *160*, 181–217, doi:10.1016/0022-2836(82)90174-7.
14. Lah, M.S.; Dixon, M.M.; Pattridge, K.A.; Stallings, W.C.; Fee, J.A.; Ludwig, M.L. Structure-Function in Escherichia Coli Iron Superoxide Dismutase: Comparisons with the Manganese Enzyme from Thermus Thermophilus. *Biochemistry* **1995**, *34*, 1646–1660, doi:10.1021/bi00005a021.
15. Ko, T.-P.; Safo, M.K.; Musayev, F.N.; Di Salvo, M.L.; Wang, C.; Wu, S.-H.; Abraham, D.J. Structure of Human Erythrocyte Catalase. *Acta Cryst D* **2000**, *56*, 241–245, doi:10.1107/S0907444999015930.

16. Mitsuhashi, S.; Mizushima, T.; Yamashita, E.; Yamamoto, M.; Kumasaka, T.; Moriyama, H.; Ueki, T.; Miyachi, S.; Tsukihara, T. X-Ray Structure of β-Carbonic Anhydrase from the Red Alga,Porphyridium Purpureum, Reveals a Novel Catalytic Site for CO2 Hydration *. *Journal of Biological Chemistry* **2000**, *275*, 5521–5526, doi:10.1074/jbc.275.8.5521.
17. Wu, R.; Gu, M.; Wilton, R.; Babnigg, G.; Kim, Y.; Pokkuluri, P.R.; Szurmant, H.; Joachimiak, A.; Schiffer, M. Insight into the Sporulation Phosphorelay: Crystal Structure of the Sensor Domain of Bacillus Subtilis Histidine Kinase, KinD. *Protein Science* **2013**, *22*, 564–576, doi:10.1002/pro.2237.
18. Zeglis, B.M.; Pierre, V.C.; Kaiser, J.T.; Barton, J.K. A Bulky Rhodium Complex Bound to an Adenosine-Adenosine DNA Mismatch: General Architecture of the Metalloinsertion Binding Mode. *Biochem.* **2009**, *48*, 4247–4253.
19. Pjura, P.E.; Grzeskowiak, K.; Dickerson, R.E. Binding of Hoechst 33258 to the Minor Groove of B-DNA. *Journal of Molecular Biology* **1987**, *197*, 257–271.
